# Disordered Rocksalts as High‐Energy and Earth‐Abundant Li‐Ion Cathodes

**DOI:** 10.1002/adma.202502766

**Published:** 2025-05-06

**Authors:** Han‐Ming Hau, Tucker Holstun, Eunryeol Lee, Bernardine L. D. Rinkel, Tara P. Mishra, Max Markuson DiPrince, Rohith Srinivaas Mohanakrishnan, Ethan C. Self, Kristin A. Persson, Bryan D. McCloskey, Gerbrand Ceder

**Affiliations:** ^1^ Department of Materials Science and Engineering University of California Berkeley Berkeley CA 94720 USA; ^2^ Materials Sciences Division Lawrence Berkeley National Laboratory Berkeley CA 94720 USA; ^3^ Department of Chemical and Biomolecular Engineering University of California‐Berkeley Berkeley CA 94720 USA; ^4^ Energy and Distributed Resources Division Lawrence Berkeley National Laboratory Berkeley CA 94720 USA; ^5^ Bredesen Center for Interdisciplinary Research and Education University of Tennessee Knoxville Knoxville TN 37996 USA; ^6^ Chemical Sciences Division Oak Ridge National Laboratory Oak Ridge TN 37830 USA

**Keywords:** cathode materials, disordered rocksalt, Li‐ion batteries

## Abstract

To address the growing demand for energy and support the shift toward transportation electrification and intermittent renewable energy, there is an urgent need for low‐cost, energy‐dense electrical storage. Research on Li‐ion electrode materials has predominantly focused on ordered materials with well‐defined lithium diffusion channels, limiting cathode design to resource‐constrained Ni‐ and Co‐based oxides and lower‐energy polyanion compounds. Recently, disordered rocksalts with lithium excess (DRX) have demonstrated high capacity and energy density when lithium excess and/or local ordering allow statistical percolation of lithium sites through the structure. This cation disorder can be induced by high temperature synthesis or mechanochemical synthesis methods for a broad range of compositions. DRX oxides and oxyfluorides containing Earth‐abundant transition metals have been prepared using various synthesis routes, including solid‐state, molten‐salt, and sol‐gel reactions. This review outlines DRX design principles and explains the effect of synthesis conditions on cation disorder and short‐range cation ordering (SRO), which determines the cycling stability and rate capability. In addition, strategies to enhance Li transport and capacity retention with Mn‐rich DRX possessing partial spinel‐like ordering are discussed. Finally, the review considers the optimization of carbon and electrolyte in DRX materials and addresses key challenges and opportunities for commercializing DRX cathodes.

## Graphical abstract

Disordered rocksalts with lithium excess (DRX) offer a new direction in Li‐ion cathode design beyond conventional Ni‐ and Co‐based materials. This review highlights the design principles, synthesis strategies, and performance optimization of DRX oxides and oxyfluorides for energy‐dense, sustainable batteries using Earth‐abundant transition metals and tailored cation disorder.

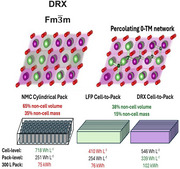

## 1 Introduction

Li‐ion batteries (LIBs) are currently the dominant electrochemical energy storage technology. Layered materials, including LiCoO_2_ and LiNi_x_Co_y_Mn_z_O_2_, have been the primary commercialized cathode chemistry, enabling the widespread adoption of personal electronics, electric vehicles, and grid‐scale storage of renewable electricity. With LIB production projected to reach 5–10 TWh / year by 2030,^[^ ^1^, ^2^ ^]^ reliance on specific transition metals (TMs), especially Cobalt (Co) and Nickel (Ni), can create risks to supply chains. Cobalt is geographically concentrated in the Democratic Republic of the Congo and is relatively expensive compared to the other 3*d* transition metals.^[^ ^3^ ^]^ While the amount of Co required per kWh of battery storage has been significantly reduced by utilizing Ni‐rich cathodes, Ni production is becoming concentrated in just a few countries in Southeast Asia, and CO_2_ production associated with its refining and mining is increasing due to the use of lower grade ore bodies.^[^ ^4^ ^]^ The singular reliance of the LIB industry on Co and Ni as redox‐active elements for high‐energy cells, and the lower energy density of LiFePO_4_ (LFP) cathodes, has recently rekindled interest in the development of energy‐dense cathode materials that use more abundant and less expensive TMs, such as iron (Fe), manganese (Mn), and chromium (Cr).

Among the dense rocksalt structures, the development of disordered rocksalt materials with Li‐excess (DRX) about a decade ago has in particular lifted the constraints on the use of specific TMs.^[^ ^5^ ^]^ DRX compounds have a crystalline rocksalt structure, where anions occupy the 4b octahedral sites on a fcc sublattice, and the cations randomly occupy the 4a octahedral sites of the interpenetrating fcc lattice (and some tetrahedral interstitials of this cation framework).^[^ ^5^, ^6^, ^7^, ^8^ ^]^ The lack of differentiation between cation sites leads to the absence of additional diffraction features in a XRD spectrum, leaving only those of the underlying rocksalt lattice. Unlike layered structures, which require Ni or Co to stabilize the structure, the cation disorder in DRX allows incorporation of a large variety of TMs. Various reports have shown high energy density and discharge capacity achieved in DRX materials using Ni, Fe, V, Mn, Ti, Cr, Nb, and Mo.^[^ ^9^, ^10^, ^11^, ^12^, ^13^, ^14^, ^15^, ^16^, ^17^, ^18^ ^]^ Compositions based primarily on Mn and Ti, whose precursors are about 15 and 80 times less expensive than Co and are orders of magnitude more abundant than Co or Ni,^[^ ^7^ ^]^ are of particular interest as Earth‐abundant cathode materials. Mn and Ti are present over a wide geographic distribution, with mainland China, South Africa, Australia, and Canada being the leading producers.^[^ ^19^ ^]^ Therefore, the possibility of using a wider range of TMs, while maintaining high performance, make DRX cathodes promising for alleviating the cost and supply chain issues of energy‐dense LIBs. In contrast to other Earth‐abundant options such as polyanion LFP and Li(Fe,Mn)PO_4_ (LMFP), DRX cathodes have higher specific energy and higher crystal density, leading to higher energy density. Like the stable Fe^+2^/Fe^+3^ redox in polyanion materials, Mn‐based DRXs maintain a stable TM ion (Mn^4+^) in the charged state, which can potentially lead to cell‐to‐pack (CTP) benefits.^[^ ^20^ ^]^ As DRX materials are at an earlier stage of development compared with layered cathodes and LFP, the combination of high energy density, Earth‐abundance, and safety has yet to be widely exploited. In this review, we summarize the basic science of DRX compounds and highlight recent accomplishments in improving their performance.

**Figure 1:**
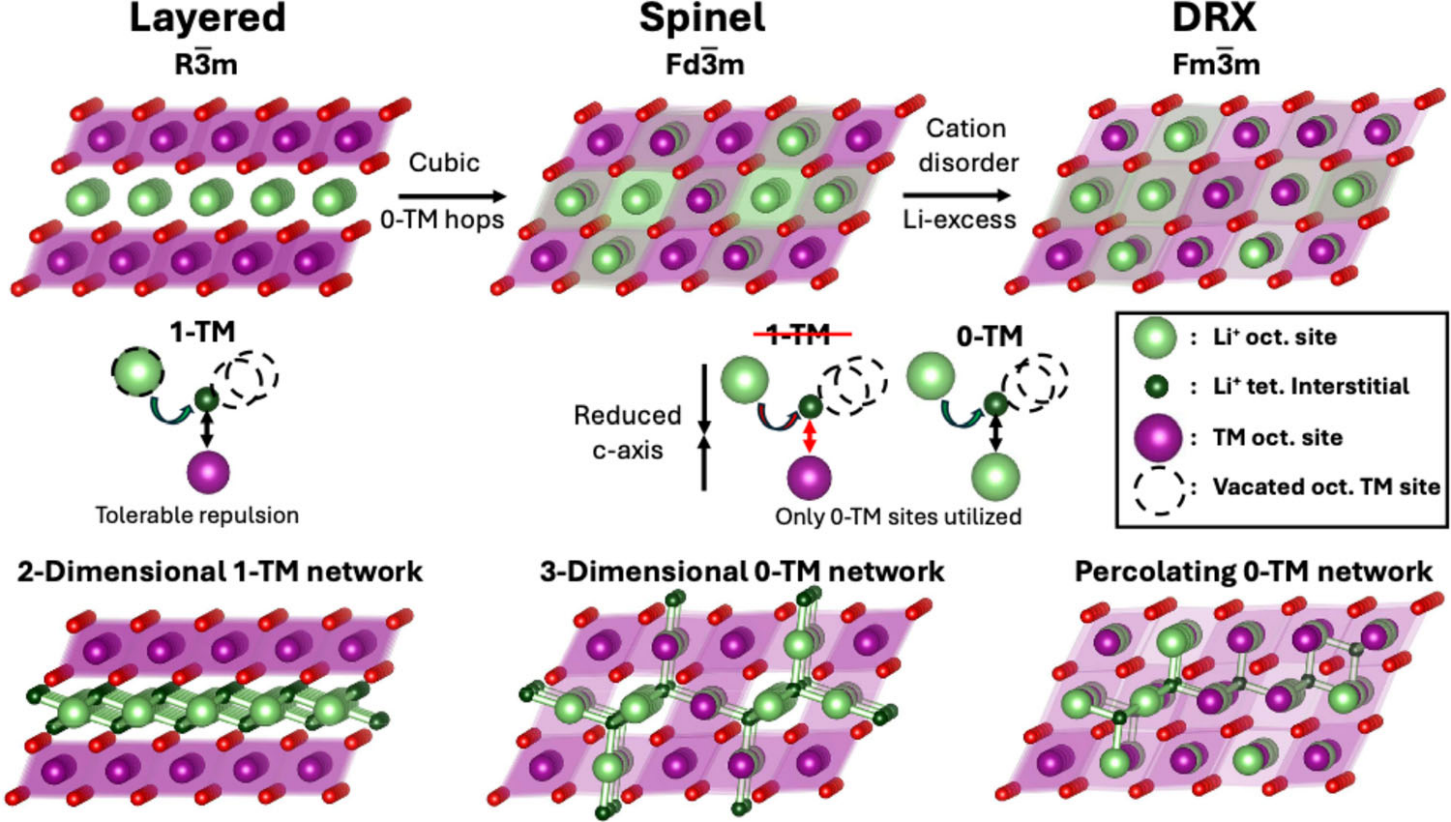
Overview of DRX and ordered rocksalt structures and their diffusion mechanisms. Diffusion networks of layered, spinel, and DRX ordering on the same rocksalt framework.

The comparison of crystal structures and Li transport mechanism is summarized in **Figure** **1**. In layered cathodes *(R‐3* *m*), oxygen occupies the FCC lattice, and the cations (Li and TM) occupy the octahedral sites, with the Li and TM octahedra order in alternating (111) planes. Li in layered structures migrate between octahedral sites via an intermediate tetrahedral site (o‐t‐o diffusion) through a di‐vacancy mechanism,^[^ ^5^, ^8^, ^21^, ^22^ ^]^ The migration barrier of this hop is determined by the number of TM that face‐share with the intermediate tetrahedral sites. In the ordered layered structure, only 1‐TM (1 TM and 3 Li face‐share with the tetrahedral site) and 3‐TM (3 TM and 1 Li face‐share with the tetrahedral site) environments are present, but the 1‐TM channels form a 2‐D percolation network. With a typical slab distance of 2.6–2.7 Å, the migration barrier is below 500 meV for layered structures.^[^ ^8^ ^]^ Spinel structures (*Fd‐3* *m*) present an ideal cation ordering in FCC framework, as the TM occupy on the 16d octahedral sites creates 0‐TM, 2‐TM, and 4‐TM environments.^[^ ^23^ ^]^ The intrinsic 0‐TM tetrahedra provides low barrier 3‐D percolation channels in spinel structures, making spinel a high‐rate cathode materials. With the same FCC framework, DRX (*Fm‐3* *m*) has no long‐range cation ordering, creating all possible tetrahedral sites with 0‐TM, 1‐TM, 2‐TM, 3‐TM, and 4‐TM as face‐sharing neighbors.^[^ ^5^, ^6^, ^7^, ^8^ ^]^

Similar to layered structures, where Li migrates through a di‐vacancy mechanism, the migration energy depends on the number of TMs present in the octahedra that face‐share with the tetrahedral site. Among these, only 0‐TM and 1‐TM are considered active, as the other tetrahedral sites have too strong an electrostatic repulsion between Li and face‐sharing TM for Li ions to migrate through them. Therefore, a percolating network of octahedral sites linked by 0‐TM and 1‐TM channels is required for Li to diffuse through DRX.^[^ ^24^ ^]^ In early DRX work, where disordered materials were synthesized without Li‐excess, low capacity was typically obtained.^[^ ^25^, ^26^, ^27^, ^28^, ^29^ ^]^ Monte–Carlo percolation simulations have shown that for a fully random distribution of cations, at least 9% of Li‐excess is required to achieve a percolating network of 0‐TM sites.^[^ ^5^, ^8^ ^]^ Experimental reports on DRX with high capacity also corroborate these theoretical estimations and 10–20% Li‐excess level is typically used for DRX materials,^[^ ^30^, ^31^, ^32^, ^33^, ^34^ ^]^ though less Li excess is needed for nano‐sized materials with very short diffusion lengths and for some recently reported partially ordered materials.^[^ ^35^, ^36^, ^37^, ^38^ ^]^ Short‐range cation ordering (SRO), in which the cation occupancies deviate from being truly random on a very short length scale, can be substantial in DRX materials.^[^ ^39^, ^40^, ^41^, ^42^, ^43^, ^44^ ^]^ While SRO can take on many types, most SRO types typically found in DRX materials tend to reduce the percolation of 0‐TM channels.^[^ ^41^, ^45^ ^]^ More recently, the diffusion theory for DRX materials has been revised to account for the influence of site energy disorder.^[^ ^46^ ^]^ It was found that variation in the lithium site energies brought about by disorder can significantly affect the energy barrier, resulting in a reduction of the lithium diffusivity by one or two orders of magnitude. This underscores the importance of controlling SRO during processing, as it may reduce site energy variation and help explain the improvements observed in partially disordered materials.^[^ ^47^ ^]^

With high compositional flexibility, DRX can incorporate many different redox‐active TM, including Fe, V, Cr, Ni, Mo, and Mn. Though Fe is the most abundant redox‐active TM, Fe‐based DRX materials have so far demonstrated limited performance. When cycled at low rates (C/60), Fe‐based DRX prepared via solid‐state synthesis delivers ≈150 mAh g^−1^ at an average voltage of 2.7 V versus Li/Li^+^, corresponding to an energy density of just 400 Wh kg^−1^.^[^ ^48^ ^]^ Mechanochemically synthesized Fe‐based DRX has shown capacity up to 292 mAh g^−1^ (700 Wh kg^−1^) between 1.3 and 4.8 V versus Li/Li^+^,^[^ ^17^ ^]^ however, the highly covalent interaction between Fe^+4^ and oxygen has made it challenging to utilize the Fe^+3^/Fe^+4^ redox couple due to its tendency to facilitate oxygen oxidation. Multiple reports have investigated Fe^+4^/O(2p) redox and attributed the large hysteresis in Fe‐based DRX to it.^[^ ^17^, ^48^, ^49^ ^]^ The mechanistic origin of the hysteresis, however, is unclear. One finding suggests that the oxygen loss followed by the structural reconstruction lead to the hysteresis, which is consistent with the low amount of Fe^+4^ observed during electrochemical cycling.^[^ ^17^, ^48^ ^]^ Recently, Li et al., proposed that Fe^+3^/O^−^ follows a kinetically favored non‐equilibrium path to Fe^+4^/O^−2^ that undergoes both Fe and oxygen redox, and attributed the large hysteresis to the Jahn–Teller activity of Fe^+4^, though at this point it is unclear by which mechanism a Jahn–Teller distortion would lead to hysteresis.^[^ ^18^, ^50^ ^]^ To enable high‐performance Fe‐based DRX cathodes, key challenges include: i) efficiently utilizing the Fe^+3^/Fe^+4^ redox couple and/or ii) stabilizing reversible oxygen redox. Given that other low‐cost TMs such as Mn are available and Fe lacks thermal stability when oxidized to the Fe^+4^ state, Fe‐based DRX is a less promising research direction at this point.

In contrast to Fe, widely available and low‐cost Cr has shown excellent performance as a redox active element in DRX compounds. The availability of a three‐electron redox couple, Cr^+3/+6^ enables co‐doping with other (inactive) stabilizing elements or the use of a large amount of Li excess (which tends to be beneficial for rate performance) while still using primarily TM redox.^[^ ^12^ ^]^ Lee et al. demonstrated that layered Li_1.211_Mo_0.467_Cr_0.3_O_2_, which electrochemically disorders to DRX after the first few cycles, maintains a capacity of 266 mAh g^−1^ with cycling.^[^ ^5^ ^]^ The use of Cr as a redox couple can also result in low or zero strain cathode materials, as only t_2__g_ electrons participate in the redox process.^[^ ^51^ ^]^ In addition, Huang et al. reported a specific mechanism by which Cr‐doping in Li_1.2_Mn_0.2_Cr_0.2_Ti_0.4_O_2_ improves performance. The oxidation of Cr^+3^ to Cr^+6^ causes a reversible octahedral to tetrahedral migration which increases the amount of available 0‐TM channels, thereby enhancing the rate capability.^[^ ^12^ ^]^ However, it is challenging to incorporate a high Cr content in DRX materials (Cr level >0.2 /f.u.) due to the strong driving force of Cr^+3^ to form a layered structure.^[^ ^52^ ^]^ In addition, the toxicity of Cr^+6^ would require careful closed‐loop management of Cr‐based DRX materials and end‐of‐life cells.

Ni has also been used as the redox‐active TM in several DRX compounds. Ni‐based DRX can be synthesized in air at low temperature (650–800 °C), lowering the energy input and cost for synthesis.^[^ ^53^ ^]^ In addition, when cycled between 1.5 and 4.8 V versus Li/Li^+^, discharge capacities of 200 mAh g^−1^, corresponding to 750 Wh kg^−1^, have been demonstrated with Ni‐based materials obtained from solid‐state and mechanochemical synthesis.^[^ ^16^, ^54^, ^55^, ^56^ ^]^ However, it is challenging to use the full Ni^+2^/ Ni^+4^ two‐electron redox couple as its electronic states overlap with oxygen states, similar to but less severely so than Fe^+3^/Fe^+4^, leading to partial oxygen oxidation when trying to access Ni^+4^ in DRX. For this reason, possibly correlated to their lower capacity, Ni‐based DRX has been less widely investigated. Stable redox activity and better retention by combining Ni with other redox‐active TMs or coating approaches are required to enhance the capacity and retention of Ni‐based DRX. Given that they would compete for the same limited Ni resources needed for high‐Ni layered cathodes, these materials would also have to demonstrate superior performance to become of interest.

Among the potential TM redox centers, Mn has shown great compatibility with the DRX structure. Mn precursors are inexpensive, and some Mn‐based DRX formulations enable double redox from Mn^+2^ to Mn^+4^. Furthermore, the fully charged Mn^+4^ state is very stable in air at room temperature.^[^ ^57^ ^]^ When using Mn^+3^/Mn^+4^ redox, Mn‐based DRX can deliver more than 250 mAh g^−1^ discharge capacity when cycled between 1.5 and 4.8 V versus Li/Li^+^.^[^ ^36^, ^58^ ^]^ Capacities > 300 mAh g^−1^, and specific energies > 900 Wh kg^−1^ have been achieved by utilizing the two‐electron Mn^+2^/Mn^+4^ redox in ball‐milled high F‐DRX.^[^ ^11^, ^15^, ^58^, ^59^, ^60^, ^61^ ^]^ While these high‐capacity DRX compounds show the potential to surpass the performance of traditional layered cathodes, a considerable amount of work remains to be done to pair them with suitable electrolytes that are stable under strong oxidizing conditions (e.g., > 4.4 V vs Li/Li^+^) and improve capacity retention. For this reason, recent focus has been on Mn‐rich DRX (Mn > 0.6/f.u.).^[^ ^36^, ^62^ ^]^ Though these materials have more moderate capacities, they can be synthesized with classical solid‐state synthesis from common precursors and have demonstrated good capacity retention during extended cycling.^[^ ^36^ ^]^ Their specific energy and energy density are typically higher than that of LFP and comparable to low Ni‐content NMC (i.e., NMC‐111) and NMC‐532.^[^ ^63^ ^]^ Some of this performance in Mn‐rich DRX is attributed to the structural transformation that occurs when they are cycled. More specifically, these materials transform to a partially disordered, spinel‐like nano‐structured phase (δ‐phase) leading to enhanced rate capability and energy density.^[^ ^36^, ^37^, ^38^, ^62^, ^64^, ^65^ ^]^ Unlike in Li‐excess NMC materials, no voltage fade has so far been observed with cycling. The high‐performance of these compounds, coupled with the low cost and thermal stability of Mn^+4^, makes Mn‐rich DRX, referred to as δ‐DRX, the leading DRX materials class for potential commercialization.^[^ ^65^, ^66^ ^]^

**Figure 2:**
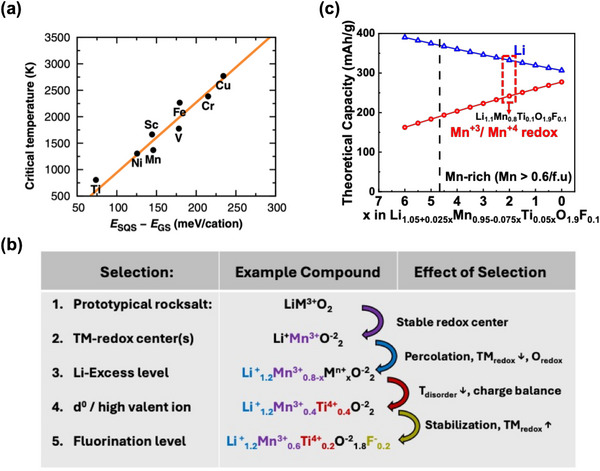
Designing a DRX. a) Predicted disordering temperature of LiMO2 species. Reproduced with permission.^[^ ^69^ ^]^ Copyright 2016, Wiley‐VCH. b) Schematic showing the design process for a DRX in the Li‐Mn‐Ti‐O‐F chemical space starting from the prototypical rocksalt LiMO2. c) Trade‐off between Li‐excess level and Mn redox capacity for Mn‐rich oxyfluorides. oxyfluorides.

Besides the redox active transition metal, other compositional elements play a role in both the performance and synthesizability of DRX materials. DRX can be best described with the general formula Li_1+y_M_r_M_d_O_2‐x_F_x_ where y is the Li excess and y + r + d = 1. The role of Li excess is well documented in the literature. Higher Li‐excess tends to improve rate capability but can reduce cycle life by both activating and requiring more oxygen redox.^[^ ^5^, ^8^, ^67^ ^]^ M_r_ denotes redox‐active TMs, and M_d_ is a high valent fully oxidized d^0^ metal (M^≥+4^) which is redox‐inactive and plays the dual role of providing charge compensation for the Li‐excess and facilitating cation disorder. Because d^0^ metals have no occupied *d*‐states, they can easily accommodate distortions from surrounding anion octahedra and facilitate the formation of cation‐disordered rocksalts.^[^ ^68^ ^]^ As can be seen in **Figure** **2**a, while most stoichiometric LiTMO_2_ compounds have high order/disorder temperatures (> 1300 °C),^[^ ^69^ ^]^ the addition of a d^0^ TM can lower the critical temperature to less than 1000 °C for most compositions, making disordered rocksalts accessible with solid‐state synthesis. Typical DRX uses 3d and 4d block d^0^ ions: Ti^+4^, Zr^+4^, Nb^+5^, and Mo^+6^.^[^ ^45^, ^49^, ^54^, ^70^, ^71^, ^72^, ^73^, ^74^ ^]^ DRX materials can also be produced without the presence of d^0^ metals, but this requires extensive ball milling due to the high disordering temperature.^[^ ^11^, ^13^, ^14^, ^32^, ^75^, ^76^ ^]^ While no detailed mapping of composition to order/disorder temperature exists, it is reasonably well understood how the presence of some cations increases it (e.g., Co^3+^, Cr^3+^, Mn^4+^) while others decrease it (mostly fully oxidized d^0^ metals). The order/disorder temperature is not the only important variable, as different cations can also influence the SRO which has a pronounced effect on Li transport. For example, ions with a similar ionic radius to Li tend to create SRO that destroys 0‐TM migration channels thereby decreasing Li transport.^[^ ^45^ ^]^ An unusual aspect of DRX materials is their ability to have part of the oxygen anions substituted by fluorine, typically performed by adding LiF to the synthesis. Typical F contents are 0≤ x≤ 0.2 for solid‐state synthesized material and 0≤ x≤ 0.66 for ball‐milled products, though the maximum F content achievable for a given compound is highly dependent on the transition metal composition. F is predicted to have mild segregation to the surface in synthesis and enhances surface protection against oxidation.^[^ ^77^ ^]^ In addition, F lowers the average anion valence enabling higher transition metal redox or higher Li excess. The role of F will be discussed in more detail in Section 3 of this review.

Charge balance and stoichiometric balance are the primary constraints in the design of DRX compositions. The excess Li required for diffusion percolation necessitates an increase in the average valence of the remaining ions. As shown in Figure 2b, a Li‐level of 1.2 (20% excess) requires the substitution of 0.4 M_r_^+3^ ions with 0.4 higher valent M_d_^+4^ (or 0.2 M_r_^+3^ with 0.2 M_d_^+5^). Alternatively, F substitution can be used to decrease the average cation valence, while also helping to stabilize the anion redox to improve the capacity retention. Not only are high valent TM charge compensators and fluorination required for charge balance, the balance between Li excess level and content of redox‐active TMs also affects the available redox in DRX as shown in Figure 2c for a material with a fixed F anion content of 5%. As the Li excess increases, the amount of Ti increases to charge‐compensate, and the available redox capacity provided by Mn decreases. For this reason, high Li‐excess DRX materials often rely on substantial oxygen redox to achieve high capacity, unless a large amount of F can be incorporated in the structure. For example, Li_2_Mn_2/3_Nb_1/3_O_2_F (Li_1.33_Mn^2+^_0.44_Nb^5+^_0.22_O_1.33_F_0.66_) has a theoretical capacity based on Mn^2+/4+^ redox of 270 mAh g^−1^, while its unfluorinated counterpart, Li_2_Mn_2/3_Nb_1/3_O_3_ (Li_1.33_Mn^3+^_0.22_Mn^4+^_0.22_Nb_0.22_O_2_) has only ≈70 mAh g^−1^ of Mn^3+/4+^ redox.^[^ ^59^ ^]^ In addition, the selection of Li, M_r_, M_d_, and F substitution level can alter the SRO and percolation network, which affects the rate capability and energy density of the materials.^[^ ^58^, ^78^ ^]^ These effects will be discussed in more detail in Sections 3 and 4.

## 2 Synthesis

Since the demonstration by Lee et al. that layered Li_1.211_Mo_0.467_Cr_0.3_O_2_ transformed to a disordered rocksalt within the first 10 galvanostatic cycles but retained high reversible capacity,^[^ ^5^ ^]^ significant efforts have been made to synthesize the cation‐disordered phase directly. These efforts include solid‐state synthesis,^[^ ^12^, ^15^, ^16^, ^41^, ^79^, ^80^, ^81^ ^]^ mechanochemical synthesis,^[^ ^10^, ^50^, ^54^, ^59^, ^82^ ^]^ molten salt synthesis,^[^ ^44^, ^65^, ^83^ ^]^ and microwave‐assisted synthesis.^[^ ^84^, ^85^ ^]^ Since the DRX phase is a high‐temperature phase that exists above the order‐disorder transition temperature of ordered rocksalts, synthesizing the DRX phase typically elevated reaction temperatures (>900 °C) or a mechanochemical approach. The variables that control the order‐disorder temperature are not fully known, but generally, a larger amount of the d^0^ element and a lower amount of non‐d^0^, redox‐active TMs make it easier to disorder the material. In this section, we will address the key aspects of various synthesis methods for DRX materials, discussing fundamental concepts, advantages, limitations, and prospects for developing advanced DRX materials.

**I) Solid‐state synthesis**. Solid‐state synthesis is the most direct and scalable approach to synthesizing DRX compounds. One must only surpass the cation disordering temperature at a given composition to stabilize DRX in synthesis. The straightforward nature of this synthesis makes it suitable for scale‐up and ensures more consistent experimental results. Many DRX materials with different TM compositions have been synthesized between 900–1100 °C under an Ar atmosphere with a sintering time of 1–6 h through solid‐state synthesis. For instance, DRX compounds with metal combinations such as Mn‐Ti,^[^ ^41^, ^80^ ^]^ Mn‐Nb,^[^ ^15^, ^81^ ^]^ Ni‐Ti,^[^ ^12^, ^86^ ^]^ V‐Nb,^[^ ^51^ ^]^ Mn‐Ti‐Cr,^[^ ^12^ ^]^ and Fe‐Nb^[^ ^87^ ^]^ have been demonstrated. Some TM compositions, such as Ni‐Ti‐Mo can be synthesized at temperatures as low as 750 °C.^[^ ^16^ ^]^ These examples illustrate the broad applicability of solid‐state synthesis for DRX materials. Because of its scalability and industrial relevance, most attention is now focused on DRX materials that can be produced by solid‐state synthesis. While these materials do not possess extremely large specific energies (≈ 1000 Wh kg^−1^) that have been demonstrated in some ball‐milled materials, they can reach specific energies that surpass LFP and comparable to low Ni‐content NMC materials. However, despite its many advantages and broad applicability, solid‐state synthesis presents challenges in controlling particle size distribution. Literature reports have reported a wide particle size distribution in the range of 10–25 µm for the as‐synthesized powders.^[^ ^88^ ^]^ Given that particle size control is critical for optimizing electrode design, density, and full‐cell performance, addressing this size distribution issue is essential in improving the solid‐state synthesis process.^[^ ^89^ ^]^

**II) Mechanochemical synthesis**. The mechanochemical approach is widely applied to synthesize DRX as it is a relatively straightforward way to incorporate elements and create cation disorder.^[^ ^10^, ^50^, ^54^, ^59^, ^83^ ^]^ This synthesis involves the use of high‐energy planetary ball milling, where significant mechanical shear forces are applied to homogenize the precursors. In general, the energy and shear forces generated during ball milling facilitate the formation of metastable compositions with high disordering temperatures that cannot be achieved by conventional solid‐state synthesis.^[^ ^90^ ^]^ This characteristic has enabled exploring a wide range of compositions, expanding the chemical space of DRX materials.^[^ ^10^, ^50^, ^54^, ^59^, ^82^ ^]^ Additionally, mechanochemical synthesis is capable of introducing high fluorine content into DRX materials beyond the thermodynamic solubility limit. In traditional solid‐state synthesis, the solubility limit of LiF in the DRX phase is typically around 10% (or stoichiometry of Li_1+x_TM_1‐x_O_1.8_F_0.2_) under standard experimental conditions (*≈*1100 °C)^[^ ^91^ ^]^ though the solubility of F in DRX materials with high Mn content is likely to be lower.^[^ ^85^ ^]^ However, mechanochemical synthesis allows for much higher fluorine content, 30% (i.e., Li_1+x_M_1‐x_O_1.4_F_0.6_,) or more, thereby significantly lowering the average anion and cation valence.^[^ ^59^ ^]^ For example, Lee et al. used mechanochemical synthesis to create Li_2_Mn_2/3_Nb_1/3_O_2_F in which the presence of Mn^+2^ provides double‐redox (Mn^+2^/Mn^+4^) leading to very high electrochemical capacity and specific energy.^[^ ^59^ ^]^

Mechanochemical synthesis typically yields materials with particle sizes around 50–200 nm, which enhances the specific energy, often exceeding 700 Wh kg^−1^, with some reports reaching > 900 Wh kg^−1^. Besides the obvious diffusion length decrease due to the smaller particle size, the higher rate capability of ball‐milled materials has also been attributed to the enrichment of structural defects and a potential effect on electronic conductivity.^[^ ^10^, ^50^, ^54^, ^59^, ^82^, ^92^ ^]^ Recently, this method has been extended to synthesize partially disordered materials containing both DRX and spinel‐like or layered‐like features with high energy density.^[^ ^93^, ^94^, ^95^ ^]^ Such partially disordered phases will be discussed in section 5. For Na‐based systems, mechanochemical synthesis is so far the only way to produce DRX‐like Na‐Metal‐oxides^[^ ^96^, ^97^ ^]^ as the large size difference between Na and 3*d* transition metals creates a very strong tendency to order.^[^ ^98^ ^]^ Despite these advantages, the mechanochemical approach has critical limitations in producing scalable DRX materials. The pulverized particle morphology increases the accessible surface area and contributes to the higher energy densities. However, the increased surface area also promotes side reactions with the electrolyte at high states of charge, ultimately deteriorating cycling performance.^[^ ^6^, ^99^ ^]^ In addition, mechanochemical synthesis is sensitive to physical variables such as ball type, ball size, planetary power, and batch size, leading to inconsistencies in experimental reproducibility and scalability issues. The small particle size also necessitates large amounts of carbon in the electrode, increasing side reactions and lowering cathode‐level energy density.^[^ ^100^ ^]^ These challenges hinder its application at commercial scale, and a better understanding of how the product can be controlled is required to create scalable mechanochemically synthesized materials.^[^ ^7^, ^101^, ^102^ ^]^

**III) Molten‐salt synthesis**. To obtain monodispersed DRX materials with a controllable size distribution, molten‐salt synthesis is considered one of the most effective techniques.^[^ ^44^, ^83^ ^]^ This bottom‐up approach facilitates the fabrication of a wide range of inorganic materials with tunable size, morphology, and surface characteristics. It is also an environmentally friendly, cost‐effective, and scalable method. Chen et al. first applied this method for DRX synthesis in 2018 and successfully synthesized single‐crystal Li_1.3_Nb_0.3_Mn_0.4_O_2_ DRX with an average particle size of 5–8 µm.^[^ ^83^ ^]^ A variety of molten salts, including LiCl, NaCl, KCl, CsCl, KOH, and Li_2_SO_4_, were tested as flux agents, and phase‐pure samples were only obtained with KCl (melting point = 770 °C). While micron‐sized DRX materials require post‐synthesis particle size reduction and carbon coating via shaker‐milling to enhance cycling performance, molten‐salt synthesis remains a promising approach for producing high‐quality single‐crystal DRX materials. However, a key limitation of this method is its difficulty in producing *≈*1–2 µm DRX particles, which are necessary to facilitate rapid Li^+^ transport throughout the active materials. Developing a synthesis protocol for producing micron or submicron‐DRX with molten‐salt synthesis, is important to expand the applicability of this method. There also is a need for work to reduce the salt‐to‐precursor weight ratio from the reported 2.5‐5 to increase the yield after synthesis.

**IV) Micro‐wave synthesis**. Recently, Clément et al. reported the synthesis of Li_1.2_Mn_0.4_Ti_0.4_O_2_ and Li_1.3_Mn_0.4_Nb_0.3_O_1−x_F_x_ using a microwave‐assisted process.^[^ ^84^ ^]^ Unlike conventional methods that typically require > 2 hr of reaction time under Ar atmosphere, this technique enabled the rapid synthesis of DRX within minutes under ambient conditions. The microwave‐assisted Li_1.2_Mn_0.4_Ti_0.4_O_2_ and Li_1.3_Mn_0.4_Nb_0.3_O_1−x_F_x_ exhibited atomic arrangements and electrochemical performances comparable to those synthesized through traditional solid‐state methods, demonstrating the technique's applicability for various DRX chemistries. Additionally, the synthesized particles displayed homogeneous morphologies with sizes averaging between 1–5 µm, depending on the synthesis time. This approach represents the first DRX synthesis method that does not necessitate either an extended heating time (> 1 hr) or an inert environment, offering the potential for high‐throughput screening of new DRX chemistries.^[^ ^84^, ^85^ ^]^ However, a limitation of this method lies in the difficulty of precisely controlling the reaction environment, as the temperature within the system is challenging to measure or regulate directly.

**V) Sol–gel synthesis**. In sol–gel synthesis, the metal oxide precursors used in solid‐state synthesis are replaced by formation of a metal‐organic gel, which is subsequently fired. Sol–gel allows for an atomic‐scale mixing to achieve a homogenous precursor distribution, which can lead to lower energy reaction pathways and facilitate the formation of the desired phase during annealing. The homogeneous mixing achieved through sol–gel processes enables the production of precursor powders with cont rolled morphology and homogeneous particle size distribution at relatively low temperatures (<500 °C) while ensuring high product purity and homogeneity. Due to these advantages, sol–gel synthesis has been widely explored for various lithium‐ion battery cathode materials. Notably, Patil et al. utilized this method to synthesize Li_1.2_Mn_0.4_Ti_0.4_O_2_ at 900 °C, observing that DRX formation proceeds at moderate temperature (800 °C).^[^ ^103^ ^]^ In contrast, solid‐ state synthesis requires annealing at higher temperatures (> 900 °C) for longer times (more than 9 h) to obtain the pure DRX phase. Patil's study demonstrated that sol–gel‐derived Li_1.2_Mn_0.4_Ti_0.4_O_2_ exhibited stable cycling and, in some cases, an increase in capacity upon cycling compared to those synthesized using conventional solid‐state methods. These findings underscore the importance of precursor mixing strategies, with sol–gel synthesis offering a potential pathway in DRX synthesis.

**Figure 3:**
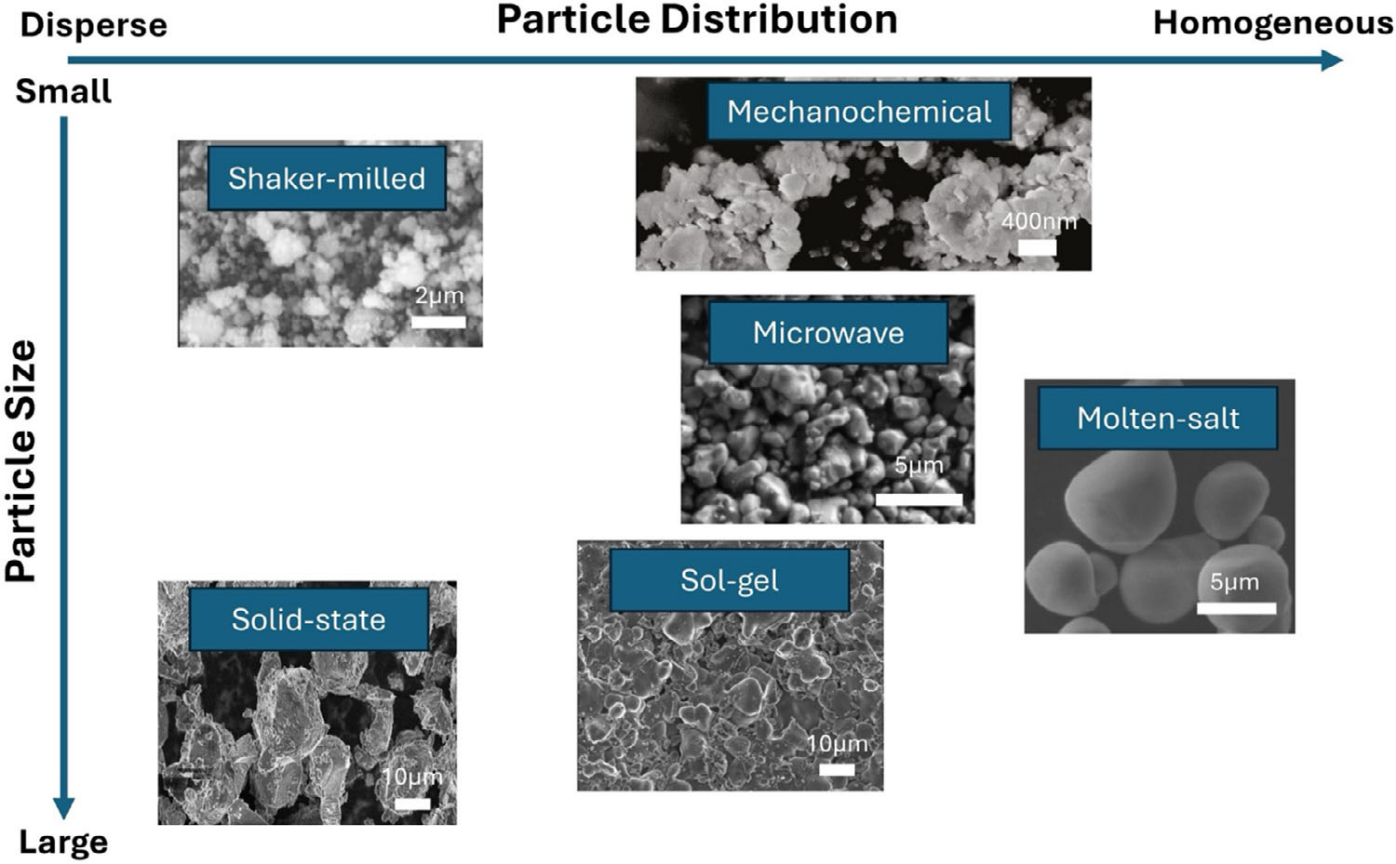
DRX Synthesis Methods. The schematic representation for various synthesis techniques for DRX materials. Each method is plotted to feature its particle distribution and average particle size. Reproduced with permission.^[^ ^9^, ^59^, ^83^, ^84^, ^88^, ^103^ ^]^ Copyright 2016, Springer Nature. Copyright 2018, Springer Nature. Copyright 2018, American Chemical Society. Copyright 2023, Wiley‐VCH. Copyright 2022, Wiley‐VCH. Copyright 2023, Wiley‐VCH.

With each method presenting unique strengths and limitations, we compare various DRX synthesis approaches based on their impact, particularly on average particle size and size distribution in **Figure** **3**. Notably, most DRX particles, regardless of the synthesis method, require post‐synthesis particle pulverization through shaker‐milling to improve rate performance due to the inherently low Li^+^ conductivity in many DRX compositions. The shaker‐milled particles typically exhibit a broad size distribution, ranging from tens to hundreds of nanometers. Similarly, mechanochemically synthesized DRXs yield particles smaller than 200 nm with a narrower size distribution, owing to the extended milling times (>20 h). However, enhancing the intrinsic conductivity of DRX materials is crucial since nanosized particles with a broad size distribution after milling adversely affect electrode‐level performance, including low volumetric density and poor capacity retention. Solid‐state synthesis typically results in large particles with a wide size distribution. Although sol–gel synthesis offers better atomic homogeneity, the precursors still require sintering, resulting in inhomogeneous particle size similar to what is found in solid‐state methods. Microwave‐assisted and molten‐salt synthesis methods produce particles with controlled size distributions, in the range of a few micrometers. However, further optimization is needed to produce smaller crystallites that ensure optimal Li‐ion transport.

Finally, recent studies have highlighted the influence of factors other than particle size and morphology, such as the local ordering (e.g., domain structures,^[^ ^62^, ^93^, ^104^ ^]^ short‐range order)^[^ ^41^, ^105^ ^]^ of the as‐synthesized DRX materials. These findings underscore the critical role of the synthesis route in optimizing material properties, beyond just the particle size and its dispersion. Thus, similar to the invention of co‐precipitation which made high‐quality NMC materials possible,^[^ ^106^, ^107^, ^108^ ^]^ advancements in synthesis techniques will be pivotal in optimizing and scaling up the production of DRX.

## 3 Fluorination

**Figure 4:**
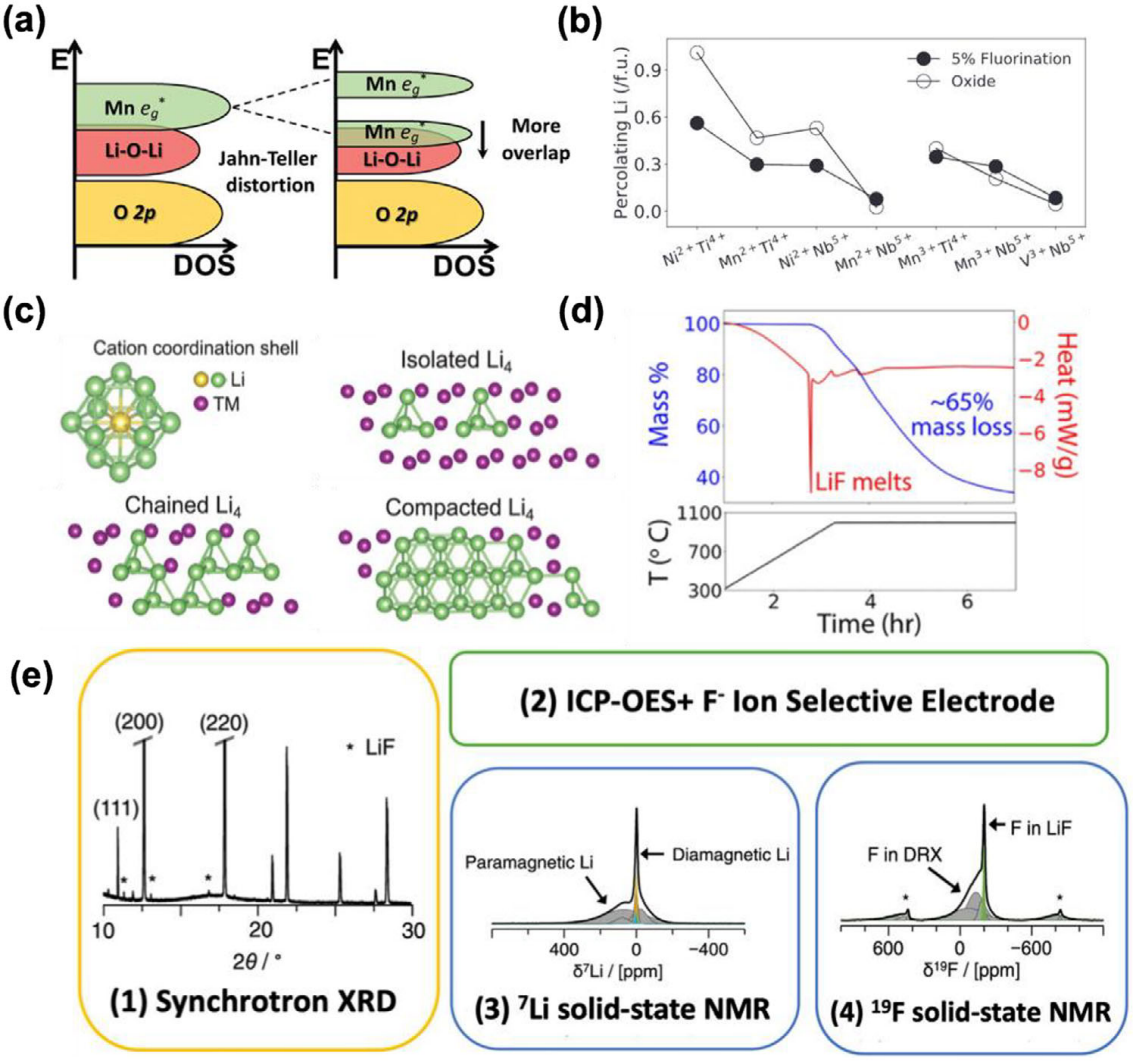
Fluorination Effects and Characterization of F in DRX. a) Effect of fluorination and orbital energy levels in Mn^+3^ system (Jahn–Teller active). Reproduced with permission.^[^ ^15^ ^]^ Copyright 2019, Wiley‐VCH. b) Comparison of the amount of 0‐TM percolating Li with and without 5% fluorination for seven DRX TM pairs with Li_1.2_/f.u. obtained from ab‐initio simulations at T = 1273 k. Reproduced with permission.^[^ ^78^ ^]^ Copyright 2020, Wiley‐VCH. c) Atomic configurations of the first neighboring cation coordination shell and examples of isolated, chained, and compacted Li_4_ (Li‐rich environments) distribution. Reproduced with permission.^[^ ^78^ ^]^ Copyright 2020, Wiley‐VCH. d) TGA/DSC measurements of LiF when annealed at 1100 °C. Reproduced with permission.^[^ ^124^ ^]^ Copyright 2022, American Chemical Society. e) Characterization methods for quantifying bulk F level in DRX. Reproduced with permission.^[^ ^80^ ^]^ Copyright 2024, American Chemical Society.

DRX materials have a unique capability to incorporate fluorine. In the traditional layered cathode compounds, substitution of O by F leads to 3 unfavorable high‐energy TM‐F bonds, resulting in virtually no equilibrium solubility for F.^[^ ^109^, ^110^ ^]^ In contrast, the Li‐excess and cation disorder in DRX create some oxygen sites with Li‐rich environments (more than 3 coordinating Li) that become preferential sites for F substitution. Fluorination has several benefits: 1) Stabilization of the material against oxygen oxidation and oxygen release, 2) Lowering of the cation valence, enabling higher theoretical TM redox capacity, and 3) Modification of the short‐range order in DRX. Stabilization of materials with oxygen redox via fluorination has been demonstrated in various DRX systems.^[^ ^13^, ^14^, ^33^, ^71^, ^111^, ^112^, ^113^, ^114^, ^115^, ^116^, ^117^ ^]^ This protection may be attributed to the F‐rich surface, which limits surface oxygen loss, and by substituting the oxygen sites most vulnerable to oxidation.^[^ ^77^, ^118^, ^119^, ^120^, ^121^ ^]^ Early theory work has established that oxygen anions surrounded by Li^+^ (Li‐O‐Li configurations) are most likely to oxidize first,^[^ ^67^ ^]^ and it is exactly these Li‐rich environments which F^−^ prefers to substitute into. The highly electronegative nature of F^−^ lowers the highest occupied molecular orbital (HOMO) level, thereby increasing the oxidation potential of the materials.^[^ ^7^, ^15^, ^59^ ^]^ Lee et al. and Lun et al. have also demonstrated that fluorination in some Mn^+3^ containing materials (Li_2_Mn_2/3_Nb_1/3_O_2_F and Li_1.2_Mn_0.65_Nb_0.15_O_1.9_F_0.1_) reduces the Jahn–Teller distortions as the symmetry breaking from the mixed O/F octahedral environment lifts the degeneracy of the Mn e_g_^*^ orbitals which is responsible for the Jahn–Teller distortion (**Figure** **4**a).^[^ ^15^, ^59^ ^]^

At fixed Li‐excess level, substituting oxygen with F^−^ lowers the average cation charge, which can be used as a strategy to increase the TM redox capacity.^[^ ^15^, ^58^, ^78^ ^]^ This has been exploited to create DRX materials with high capacity by using the two‐electron redox obtained by oxidizing from Mn^+2^ to Mn^+4^.^[^ ^59^ ^]^ The strong preference for Li‐F bond formation also modifies the SRO of the materials and changes the redox behavior. For example, in Ni‐based DRX, F preferentially surrounds itself with Li and repels Ni. This modifies the redox mechanism from a distinct Ni^+2^/Ni^+3^ and Ni^+3^/Ni^+4^ oxidation of Ni bonded to F to direct Ni^+2^ to Ni^+4^ oxidation when Ni is in Ni‐O_6_ environments.^[^ ^55^ ^]^ While F doping generally enhances the capacity retention and TM redox capacity of DRXs as shown in Figure 4b, fluorination has more complex effects on the percolating Li content of DRX materials. In compositions where percolating Li is low, it tends to improve with fluorination, while in materials with an already high content of percolating Li, it decreases with fluorination. As shown in Mn oxyfluorides with the general formula Li_x_Mn_2‐x_O_2‐y_F_y_, the higher F level in Li_1.333_Mn_0.667_O_1.333_F_0.667_ does not lead to higher initial capacity when compared to Li_1.333_Mn(III)_0.333_Mn(IV)_0.333_O_1.667_F_0.333_.^[^ ^58^ ^]^ Ab‐initio cluster expansion modeling of the configurational disorder at high temperature attributes this to the very high F content in the material, which can confine Li within small domains. This Li segregation lowers the capacity by forming isolated 0‐TM clusters instead of a percolation network as shown in Figure 4c.^[^ ^58^ ^]^ In addition, a “Li‐gettering” effect due to fluorination proposed by Kitchaev et al. was observed in both Mn‐based and V‐based DRX, where some Li cannot be electrochemically extracted at reasonable voltages due to strong Li‐F bonding.^[^ ^90^ ^]^ Although this effect may stabilize the material at high voltage, it also sets a constraint for the capacity optimization of the cathode. The trade‐off between enhanced stability and good Li percolation through F doping may vary between material systems, and the ideal amount of F substitution should be optimized carefully with the associated change in SRO and redox behavior.

The thermodynamic F solubility depends on the TM content. Higher amounts of Li and d^0^ elements lead to the highest F solubility. In contrast, high Mn content, which is relevant for the most recent δ‐DRX materials,^[^ ^36^, ^37^, ^66^, ^113^ ^]^ will almost certainly lead to lower F solubility as Mn‐F bonds are unfavorable compared to Li‐F. Early ab initio thermodynamics work indicated a F solubility in DRX of ≈10–15%.^[^ ^91^ ^]^ However, multiple reports demonstrate that higher F substitution levels can be achieved in either solid‐state or mechanochemical synthesized oxyfluorides. With the latter approach, non‐equilibrium F incorporation up to 50% has been observed.^[^ ^15^, ^58^, ^60^, ^76^, ^122^ ^]^ Care has to be taken to evaluate the F content when solid‐state synthesis is performed at high temperature above 1000 °C, which can increase the F solubility in solid‐state synthesis to 7.5%, as demonstrated experimentally in some compounds.^[^ ^85^, ^123^ ^]^ But synthesis above the melting point of LiF (848 °C) can lead to confusing results as any LiF that is not incorporated into a rocksalt compound (or other compound) above this temperature evaporates quickly, as shown in Figure 4d. This often leads to a lower F level than targeted in the DRX. Szymanski et al. studied in detail the behavior of various fluorination processes and precursors and showed that most of them convert to LiF when in contact with a Li salt.^[^ ^124^ ^]^ Because this reaction occurs at much lower temperature than the rocksalt formation, the actual F‐incorporation into DRX starts from LiF, regardless of the actual precursor used. To ensure high F uptake and limited LiF loss above 848 °C, it is important to mix the precursors well so that rocksalt compounds can form quickly and effectively lock in the F. An in‐situ TEM study has shown proof of concept for this showing rocksalt formation as quickly as a few minutes at 900 °C.^[^ ^88^ ^]^

The potential evaporation of LiF at high temperatures underscores the importance of determining F content after synthesis. However, accurately quantifying F in DRX materials presents several challenges. Due to the similar scattering lengths of O and F for both neutrons and X‐rays, these elements cannot be distinguished in diffraction experiments. Furthermore, the high volatility of LiF means that its absence in XRD patterns does not necessarily confirm that all fluorine has been incorporated, as highlighted by Szymanski et al.^[^ ^124^ ^]^ To quantify the amount of F incorporated into the bulk structure, Giovine et al. introduced a combination of synchrotron XRD, inductively coupled plasma optical emission spectroscopy (ICP‐OES), fluoride ion‐selective electrode (F‐ISE), and Li and ^19^F solid‐state NMR (ss‐NMR) to determine a lower and upper bound on the F level (Figure 4e).^[^ ^80^ ^]^ In this approach, synchrotron XRD is applied to determine the structure and identify possible impurity phase or low crystallinity LiF. The overall (DRX and impurities) elemental ratios of the samples are determined by ICP‐OES and F‐ISE. To separate the Li and F content that is present in the paramagnetic environment of the DRX, ^7^Li and ^19^F ss‐NMR are employed. The broad NMR spectrum that F in the DRX generates allows it to be distinguished from F present in LiF, where it shows a sharp diamagnetic resonance feature in the ss‐NMR spectrum. By fitting the ss‐NMR spectrum, the ratio of F and Li in the DRX to those in impurities can be obtained, and the amount of F in the bulk can be estimated by combining the results from ss‐NMR, ICP‐OES, and F‐ISE. While the combination of these various techniques can lead to a determination of F‐content, a more accessible characterization technique, which can directly measure the bulk F‐content, would help clarify the solubility limits across various compositions.

## 4 Rate Capability and Short‐Range Order

**Figure 5:**
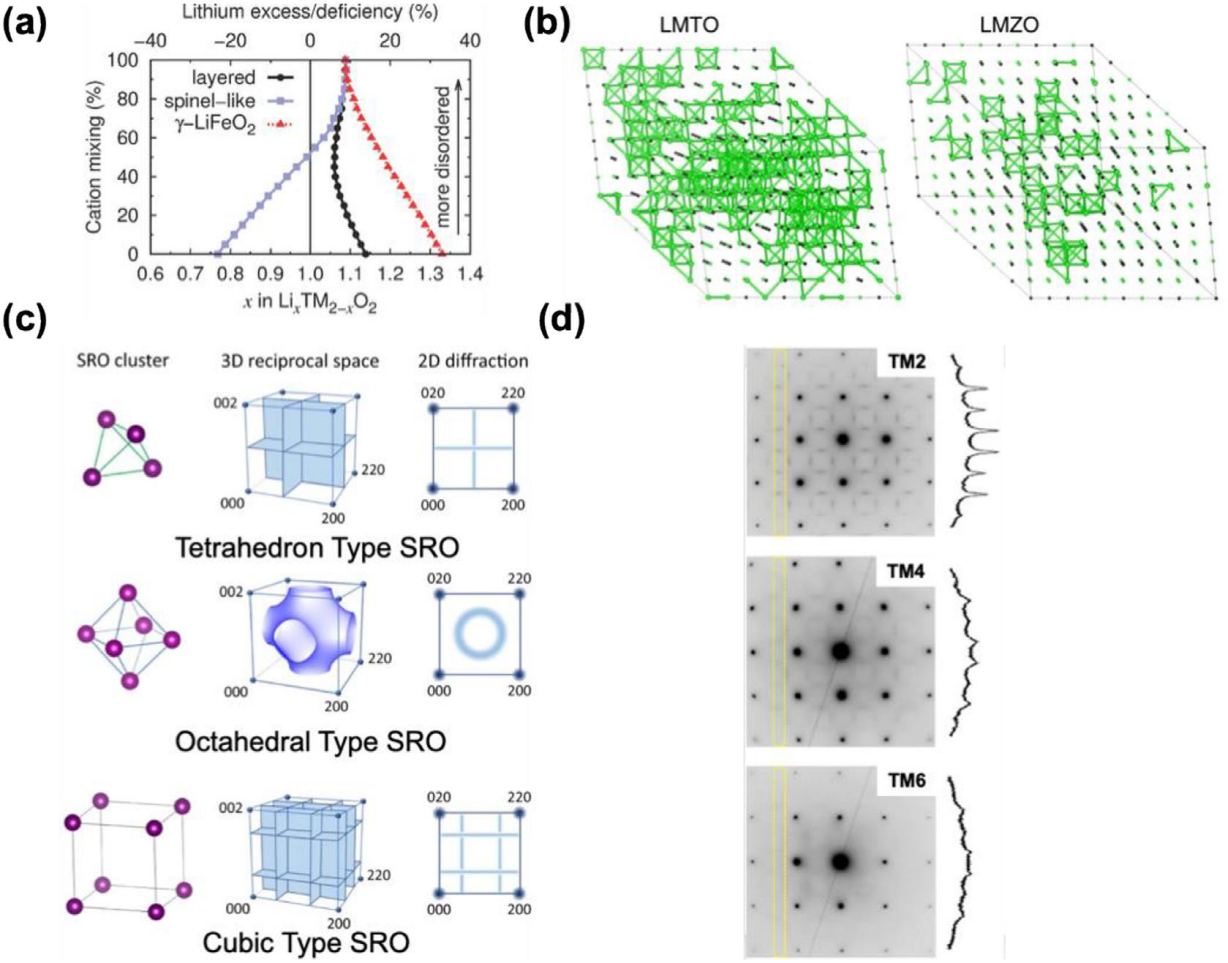
Effect of SRO in DRX cathodes. a) Critical lithium concentrations for 0‐TM percolation as a function of cation mixing level and Li level for different polymorphs. Reproduced with permission.^[^ ^8^ ^]^ Copyright 2014, Wiley‐VCH. b) Monte‐Carlo simulated structures for LMTO and LMZO, where the green spheres represent the 0‐TM channels. Reproduced with permission.^[^ ^45^ ^]^ Copyright 2019, Springer Nature. c) Basic short‐range ordering observed in DRX cathodes together with the reciprocal patterns and the 2D diffraction. Reproduced with permission.^[^ ^41^ ^]^ Copyright 2023, Springer Nature. d) TEM ED patterns along the^[^ ^100^ ^]^ zone axis from as‐synthesized DRX with 2 (TM2), 4 (TM4), or 6 (TM6) transition metal ions. The square‐like diffuse scattering patterns can be attributed to the SRO. The intensity integrated between the yellow lines is shown on the side of the ED. Reproduced with permission.^[^ ^105^ ^]^ Copyright 2021, Springer Nature.

Short‐range cation order (SRO) has been found to be a key factor in determining the rate capability of DRX materials. Its control, through tuning of the composition and synthesis, has become the most effective tool to improve Li transport in these materials. In a DRX with a truly random arrangement of cations, about 9% Li‐excess is needed to achieve percolation of Li‐sites via 0‐TM gateways as shown in **Figure** **5**a.^[^ ^8^ ^]^ However, disordered materials deviate from this truly random disorder, impacting the number of 0‐TM environments in the material without being long‐range enough to reduce the symmetry of the rocksalt lattice and create distinct cation sites. As a result, SRO can modify the percolation limit by influencing the probability of different local cation environments, and therefore the connectivity of 0‐TM sites.^[^ ^39^, ^42^, ^43^, ^125^ ^]^ Depending on the type of SRO, this can alter the Li‐excess level necessary for achieving percolation. Moreover, the local ordering around the 0‐TM pathways impacts the migration barriers, affecting Li diffusion even when percolation is achieved.^[^ ^46^ ^]^

A comparative study by Ji et al. demonstrated that Li_1.2_Mn_0.4_Ti_0.4_O_2_ and Li_1.2_Mn_0.4_Zr_0.4_O_2_ show very different discharge behavior despite having identical Li‐excess levels and available TM redox.^[^ ^45^ ^]^ That study revealed that the TM selection affects the Li‐conduction environments due to SRO as shown in Figure 5b. SRO is driven by the short‐distance elastic and electrostatic interactions. In general, metals with large ionic radius TM (Zr^+4^, Sc^+3^, and In^+3^) should be avoided, as their lack of size differentiation with lithium enables them to mix well around a tetrahedron, reducing the probability of forming 0‐TM channels. High valent ions (Ti^+4^, Zr^+4^, Nb^+5^) can also favor mixing, as local charge balance requires that they exist near Li‐rich environments. In contrast, a divalent TM does not require Li to maintain local electroneutrality,^[^ ^126^ ^]^ and smaller ions preferentially occupy smaller octahedra, with fewer neighboring Li. These effects promote Li segregation and facilitate efficient Li transport.^[^ ^45^ ^]^

While the lack of long‐range order in XRD provides no evidence of deviations from a random cation distribution, the effect of the SRO on the rate capability and capacity of DRX cathodes can be characterized through TEM and pair distribution functions (PDFs) to study ordering at this shorter length scale.^[^ ^45^, ^126^, ^127^ ^]^ In TEM electron diffraction (ED), SRO shows up as diffuse scattering intensity between the Bragg reflections. While the SRO type can be differentiated, it is challenging to quantify SRO from electron diffraction. Ji et al. used ED to show that the poor performance of Li_1.2_Mn_0.4_Zr_0.4_O_2_ (LMZO) could be explained by the pronounced tetrahedral cation SRO clusters while Li_1.2_Mn_0.4_Ti_0.4_O_2_ (LMTO), with better performance, showed an octahedral cation SRO.^[^ ^45^ ^]^ By combining TEM, ED, and ab initio cluster expansion Monte Carlo (CEMC), Li et al showed that SRO patterns can be categorized in reciprocal space into three basic types: tetrahedron, octahedron, and cubic.^[^ ^41^, ^126^ ^]^ Each SRO type has a distinct diffraction locus and diffuse scattering pattern as shown in Figure 5c. When applied to compare an unfluorinated DRX (LMTO) and a fluorinated compound Li_1.2_Ti_0.2_Mn_0.6_O_1.8_F_0.2_ (LMTOF), LMTO with the octahedral‐type SRO shows better Li percolation compared to the cubic‐type SRO in LMTOF. Therefore, careful optimization and characterization of SROs are crucial to improve the rate capability and conductivity of DRX cathodes.

Pair distribution functions (PDFs) obtained from neutron or X‐ray diffraction have been utilized to investigate short‐range order (SRO) in DRX materials. Typically, the difference between a PDF modeled on a cubic lattice with idealized random cation distribution and the experimental PDF serves as evidence for SRO. However, Szymanski et al. demonstrated that even in materials with a fully random cation environment, deviations from the “ideal PDF” can arise due to local displacements from rocksalt lattice positions. They further showed that the shapes and positions of the initial peaks in the PDF are best explained only when both (a) SRO and (b) the atomic displacements caused by SRO are accounted for.^[^ ^127^ ^]^

To minimize the formation of unfavorable sSRO, various synthesis approaches have been explored. Quenching during DRX synthesis has been shown to effectively suppress most SRO. This observation is supported by findings from an in‐situ study on DRX synthesis,^[^ ^88^ ^]^ which revealed that DRX formation without SRO occurs rapidly at high temperatures due to the significant reaction energy of the precursors. In contrast, the rearrangement of cations into SRO is a much slower process that operates on a smaller energy scale. As a result, synthesis methods that enable brief processing times at high temperatures tend to produce DRX compounds with superior rate capability and capacity. Differences in the extent of detrimental γ‐LiFeO2 type SRO formation depending on the cooling rate have been reported, with the extent of disorder in local Li environments visible in the ^7^Li NMR spectra.^[^ ^39^ ^]^ Another interesting approach is to suppress the formation of a single dominant SRO type by introducing high‐entropy in DRX (HE‐DRX).^[^ ^105^ ^]^ In high‐entropy DRX, a large number of different TMs are incorporated to increase the configurational entropy of the materials, eliminating the formation of specific SRO. Figure 5d. shows an example of decreased SRO scattering in electron diffraction as the number of TM in the compound is increased from 2 (TM2) to 4 (TM4) to 6 (TM6). Several reports indicate such SRO‐suppressed HE‐DRX cathodes show improved rate capability and can deliver high capacity.^[^ ^105^, ^128^, ^129^ ^]^

Even though most investigations of the effects of SRO on rate capability focus on the number and connectivity of 0‐TM channels, recent work has shown that the site energy disorder created by the cation disorder also contributes significantly to a reduction in Li diffusivity through the material. Anand et al. and Kang et al. showed that the variations in site energy can contribute to the Li hopping barrier and reduce the diffusion constant by up to two orders of magnitude.^[^ ^46^, ^130^ ^]^ The results further show that without this contribution, DRX compounds would have very high Li transport rate.

## 5 Partially Disordered Compounds

**Figure 6:**
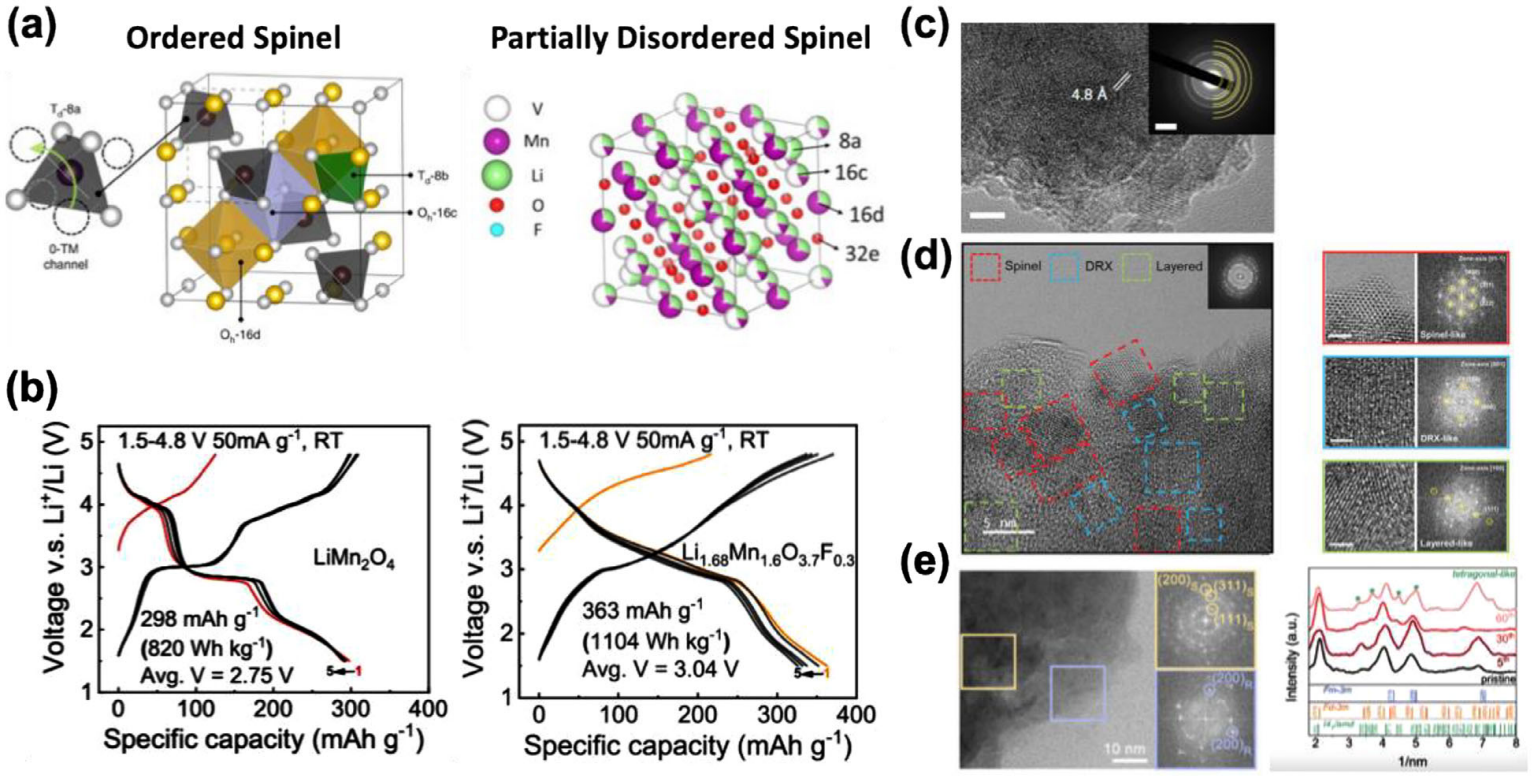
Structural and Electrochemical Properties of Partially Disordered Compounds. a) Crystal structure of ordered spinel (Left) and PDS (Right, where Td‐8a, Td‐8b, Oh‐16c, and Oh‐16d are highlighted as colored polyhedra in ordered spinel. The black, yellow, and silver spheres represent the occupying Li, M, and F/O atoms, respectively. The enlarged Td‐8a site illustrates Li migration through a 0‐TM channel in the spinel structure. In PDS, 16c sites can be occupied by Li, TM, and vacancies (V), and 16d sites can be occupied by both Li and TM, representing the 16c/16d disorder in PDS. Reproduced with permission.^[^ ^93^, ^94^ ^]^ Copyright 2023, Wiley‐VCH. Copyright 2020, Springer Nature. b) Voltage profiles of ordered spinel (Left) and PDS (Right). Reproduced with permission.^[^ ^95^ ^]^ Copyright 2020, Elsevier. c) Spinel‐type ordering from TEM ED pattern. Reproduced with permission.^[^ ^94^ ^]^ Copyright 2020, Springer Nature. d) HR‐TEM images of nanocomposite images of PDS composed of DRX, spinel, and layered phase (Left) and selected area ED patterns (Right). Reproduced with permission.^[^ ^93^ ^]^ Copyright 2023, Wiley‐VCH. e) HR‐TEM images and ED patterns of core–shell PDS with spinel and DRX phase (Left) and XRD spectrum of the growth of disordered tetragonal phase in bulk during cycling (Right). Reproduced with permission.^[^ ^104^ ^]^ Copyright 2024, Royal Society of Chemistry.

With optimized compositions and SRO, DRX compounds can demonstrate high capacity and energy density at reasonable rates. As they have a flatter energy landscape, better rate performance is expected from materials with partial order when they have local environments favorable for Li hopping. We consider here the class of materials in which some level of long‐range order exists (e.g., Bragg reflections in XRD) but with a very high degree of disorder, which distinguishes them from well‐ordered compounds with very small amounts of disorder (e.g., high Ni NMC). Such partially (dis)ordered compounds may be an ideal intermediate between well‐ordered compounds and fully disordered materials: Given enough disorder, all phase transitions of the parent ordered phase will be quenched, leading to smoother voltage profiles without problematic phase transitions. Furthermore, their partial order will also reduce the site energy variation, thereby increasing rate capability.^[^ ^46^ ^]^ While limited research exists on such partially (dis)ordered, compounds, two examples provide a proof of concept: spinel‐like and layered‐like ordering. In LiMn_2_O_4_ spinel, ab initio theory has shown that 10–20% cation disorder can transform the two‐phase region at 3 V into a solid solution, which would enable stable cycling over this extended capacity range.^[^ ^131^ ^]^ Such partially disordered spinel (PDS) materials have been created through ball milling and show very high reversible capacity.^[^ ^93^, ^94^, ^95^, ^104^, ^132^ ^]^ In PDS, both the 16c and 16d octahedral sites are partially occupied by Li or TM, and the 8a tetrahedral site has Li occupancy less than 1 (**Figure** **6**a). In a regular ordered spinel structure, disorder would lead to a significant increase in energy, as it requires either Mn^3/4+^ occupation of a tetrahedron (8a/16d disorder) or face‐sharing between 8a Li and 16c Mn (16c/16d disorder) which is energetically unfavorable. However, at a rocksalt composition, with all cations in octahedral sites, neither of these specific penalties exist. Therefore, the cation disorder in PDS is stabilized in part by the over‐stoichiometry of cations (> 3:4) relative to that of the spinel structure (3:4), which forces more ions onto octahedral 16c sites, rather than tetrahedral 8a. The voltage curve of this material, reproduced in Figure 6b, shows that the 3 V plateau is indeed absent and replaced by a solid‐solution regime, in contrast to a regular ordered spinel (left panel of Figure 6b). The 16c/16d disorder allows PDS to operate below 3 V without experiencing heterogeneous lattice strain, while utilizing the full Mn theoretical redox to achieve higher capacity compared to ordered spinel. Through its partial spinel‐like order, PDS inherits the good rate capability of regular spinel. Yang et al. also showed that 16c/16d disorder creates new Li migration pathways through the disordered spinel structure.^[^ ^133^ ^]^

Experimentally, PDS has been synthesized by mechanochemical synthesis with an average particle size of 100–300 nm. In Li_1.68_Mn_1.6_O_3.7_F_0.3_ (LMOF03) reported by Ji et al. a maximum discharge capacity of 363 mAh g^−1^, corresponding to an energy density of 1103 Wh kg^−1^, is achieved between 1.5 and 4.8 V versus Li/Li^+^ with a specific current of 20 mA g^−1^. At a higher specific current of 10 A/g, LMOF03 can still deliver > 150 mAh g^−1^.^[^ ^94^ ^]^ Aside from the high capacity and rate capability, LMOF03 suffers from capacity loss which is likely due to oxygen redox, originating from the Li‐excess environments that lead to an increase in Li‐O‐Li configurations,^[^ ^67^ ^]^ and from breakdown of the carbonate solvents in the electrolyte at the high charging voltage. Cai et al. synthesized a series of PDS materials with different extent of ordering by tuning the Li content (Li_1.4+x_Mn_1.6_O_3.7_F_0.3_) and found that a moderately disordered LMOF03 achieves the highest performance. Further increasing the Li level to Li_2.4_Mn_1.6_O_3.7_F_0.3_ results in a DRX structure with lower rate capability.^[^ ^95^ ^]^

Lee et al. proposed Ti‐doping as a strategy to stabilize PDS.^[^ ^93^ ^]^ They used ^17^O magnetic moment measurements and differential electrochemical mass spectrometry (DEMS) to argue for O‐oxidation above 4.3 V versus Li/Li^+^ and corresponding oxygen loss. Doping with Ti marginally improved the capacity retention from 53% after 50 cycles for LMOF03 to 63% and 70% respectively for Li_1.68_Mn_1.45_Ti_0.15_O_3.7_F_0.3_ (T15) and Li_1.68_Mn_1.30_Ti_0.30_O_3.7_F_0.3_ (T30).^[^ ^93^ ^]^ Alternatively, Jo et. al increased the Mn content and reduced the Li‐excess to obtain Li_1.33_Mn_2_O_4_, which achieves a 98.4% of capacity retention after 60 cycles.^[^ ^104^ ^]^ Combined, these studies confirm that a higher Li level increases the rate performance but degrades the capacity retention by increasing oxygen redox. Due to the reproducibility challenges with mechanochemical synthesis and the difficulty of characterizing partially disordered structures, different structural interpretations have been proposed for PDS. Ji et al. and Cai et al.^[^ ^94^, ^95^ ^]^ used electron diffraction to index the sample to the spinel structure, as shown in Figure 6c. Partial disorder in the spinel was further confirmed with neutron and synchrotron X‐ray diffraction. In contrast, Lee et al. proposed a nanocomposite structure as shown in Figure 6d. Their high‐resolution TEM (HR‐TEM) image shows a combination of layered, DRX, and spinel phases, though these phases may contain substantial cation disorder which would be needed to explain the voltage profile and mitigate the collective Jahn–Teller distortions.^[^ ^93^ ^]^ On the basis of HR‐TEM and electron diffraction, Jo. et al. rather proposed a core–shell composite with DRX surface and spinel‐like bulk as shown in Figure 6e. Understanding the actual structure of PDS, or to what extent variations arise from differences in ball milling, precursors etc., is important for the further development of this high specific energy materials class.

In another example of the benefits of partial disorder, Huang et al. reported that when layered Li_1.2_Cr_0.4_Mn_0.4_O_2_ was made partially disordered through ball milling, voltage hysteresis became much lower than in the ordered state. This performance improvement was attributed to disorder that prevents collective TM migrations, which is responsible for the hysteresis in the well‐ordered material.^[^ ^47^ ^]^ By using Lithium bis(fluorosulfonyl)amide (LiFSI), dimethyl carbonate (DMC) in a molar ratio of 1:1.1 as electrolyte, the dissolution of Cr can be mitigated, and the material maintains over 200 mAh g^−1^ discharge capacity between 2–4.4 V versus Li/Li^+^ after 120 cycles.

Partially disordered, long‐range ordered, compounds are a new class of cathode materials with tremendous potential to combine very high energy content with high‐rate capability. The level of disorder is a new design handle that can be used to smooth or remove phase transitions and enable the use of transition metals not previously used in Li‐ion cathodes. At this point, only mechanochemical methods have been used to produce partially disordered materials in the as‐synthesized state, and more controllable synthesis methods may be needed to understand and scale the production of these compounds.

## 6 Manganese‐Rich DRX

Much of the research on DRX compounds has now coalesced around Mn‐rich compositions, as they are the closest to achieving performance characteristics and synthesis protocols that can be accepted by industry. Mn‐based DRX formulations combine high capacity with good cycling stability and reasonable rate capability.^[^ ^15^, ^58^, ^60^, ^61^ ^]^ In DRX compounds with many of the other possible TM redox centers, the sloping voltage profile pushes some of their capacity above or below reasonable voltage cutoffs. However, the Mn^+3/+4^ redox couple in a DRX occurs on average near 3 V versus Li/Li^+^, making essentially the entire redox couple accessible within a window of 2.0–4.8 V. In addition, the low cost and high abundance of Mn‐oxide precursors, and the high thermal stability of the charged Mn^+4^ state, make the element attractive for scalable low‐cost Li‐ion technology. For these Mn^+3^‐based DRXs, Ti^+4^ has attracted the most attention as a *d* ^0^ ion, on account of the relative abundance and low cost of TiO_2_ as a precursor.^[^ ^134^ ^]^ Additionally, the relatively low disordering temperature of LiMnO_2_ relative to other ordered rocksalts means that a lower content of *d* ^0^ ions may be used for Mn‐based systems, allowing for a higher amount of Mn redox as compared to compounds with other redox active TMs.^[^ ^69^ ^]^ The class of high‐Mn content DRX compounds is typically defined as containing more than 0.6 Mn per O_2_ with the general formula Li_1+x_Mn_1−3x_Ti_2x_O_2‐y_F_y_ (x < 0.133, y< 0.2).

**Figure 7:**
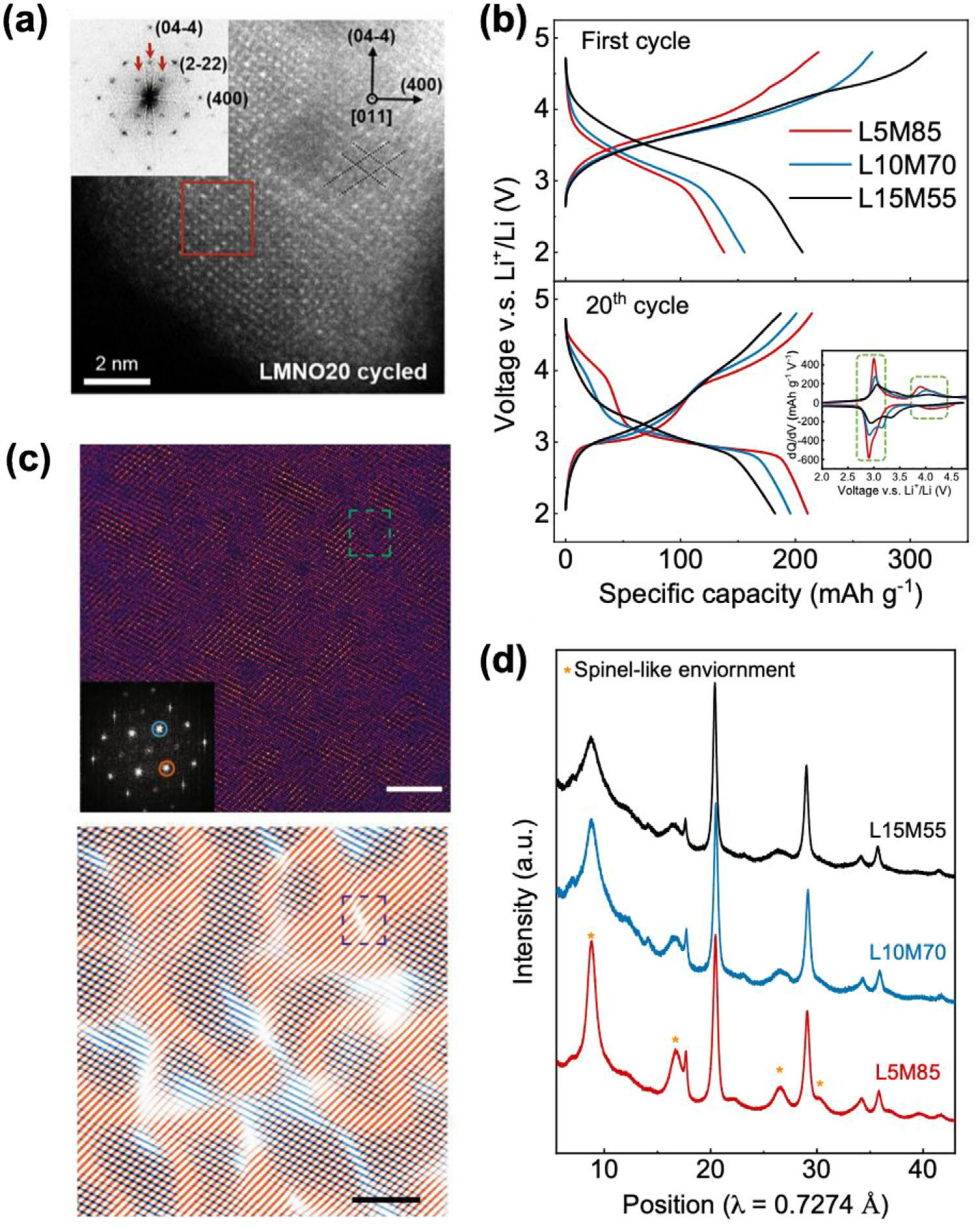
Transformation to spinel‐like δ phase in Mn‐Rich DRX a) HRSTEM images of spinel‐like environments formed in Li_1.2_Mn_0.6_Nb_0.2_O_2_) after 20 cycles, as seen down the^[^ ^110^ ^]^ zone axis. Reproduced with permission.^[^ ^64^ ^]^ Copyright 2020, Elsevier. b) Evolution of the voltage curves of L5M85(Li_1.05_Mn_0.85_Ti_0.1_O_2_), L10M70 ((Li_1.1_Mn_0.7_Ti_0.2_O_2_), and L15M55 (Li_1.15_Mn_0.55_Ti_0.3_O_2_) showing a capacity gain and the distinct formation of new features in the case of L5M85. Reproduced with permission.^[^ ^36^ ^]^ Copyright 2024, Springer Nature. c) Cation ordering from Bragg filtering for Li_1.2_Mn_0.65_Ti_0.15_O_1.9_F_0.1_. The blue dashed box in the bottom figure shows the antiphase boundary of spinel‐like environments obtained from the green square in the upper figure. Scale bar, 5 nm Reproduced with permission.^[^ ^62^ ^]^ Copyright 2024, Springer Nature. d) Ex situ synchrotron diffraction spectra of L5M85, L10M70, and L15M55, showing the development of broad, spinel‐like diffraction features in each marked with the star, but to the greatest extent in the most Mn‐rich composition (L5M85). Reproduced with permission.^[^ ^36^ ^]^ Copyright 2024, Springer Nature.

Mn‐rich DRX materials undergo significant structural and electrochemical changes during cycling. These changes enhance performance and are consistent with transformations to spinel‐like order observed in the ordered Li‐Mn‐oxides.^[^ ^135^, ^136^, ^137^, ^138^ ^]^ This transformation was first reported for Li_1.2_Mn_0.6_Nb_0.2_O_2_, in which a spinel‐like phase is formed at the surface after cycling, improving the rate capability of the material.^[^ ^64^ ^]^ Evidence of the formation of spinel‐like order can be seen in **Figure** **7**a, which shows HRSTEM of the sample after 20 cycles. Previous theory work on Mn‐rich ordered materials has shown that the spinel structure is particularly thermodynamically favorable in Mn‐based oxides when the cation composition reaches that of spinel (i.e., Li_0.5+x_M_1–x_O_2_).^[^ ^36^, ^139^ ^]^ This, taken together with the relative mobility of Mn^+2^ (and therefore Mn^+3^ by charge disproportionation to Mn^+2^ and Mn^+4^) may rationalize such transformations to spinel‐like order in Mn‐rich DRX.^[^ ^140^ ^]^ The rearrangement of Mn which occurs during this transformation to spinel‐like order leads to drastic changes to the voltage curve, with an increase in capacity and the formation of distinct plateau‐like features at ≈3 and 4 V versus Li/Li^+^, as seen in Figure 7b. This is because when cations rearrange from the random distribution in DRX to the more spinel‐like configurations, more 0‐TM sites form. In spinel space group notation, these 0‐TM environments for the 8a‐like tetrahedral sites which Li occupies at around 4 V before further lithiation moves all Li ions to the octahedral, 16c‐like sites at 3 V. While this transformed material possesses features of spinel‐like order, there are also differences in the voltage curve which indicate a clear deviation from fully ordered spinel. The content of retained disorder in these materials appears to reduce the number of available 0‐TM sites relative to an ordered spinel, leading to a shortened 4 V feature, typically contributing < 70 mAh g^−1^, whereas an ordered spinel typically delivers >100 mAh g^−1^ on its 4 V plateau.^[^ ^36^, ^38^, ^119^ ^]^ The 4 V and 3 V features in this transformed DRX also retain more slope than in a well‐ordered spinel. Due to the unique characteristic of the formed phase, it is referred to as the *δ* phase, or here as *δ*‐DRX.

Characterization of *δ*‐DRX through a combination of TEM, synchrotron XRD, and NMR suggest that these materials possess a significant degree of order, in the form of small spinel‐like “domains” separated by antiphase boundaries.^[^ ^36^, ^62^, ^66^ ^]^ The formation of domains, and antiphase boundaries can be seen in the atomic resolution HAADF‐STEM image at the top of Figure 7c, and the Fourier filtered image below. While in layered and orthorhombic LiMnO_2_ the transformation to spinel during cycling is nearly completed, resulting in highly ordered spinel,^[^ ^135^, ^136^, ^137^ ^]^ in DRX more disorder is retained in the spinel. In TEM, distinct “domains” of spinel‐like order can be observed. In complimentary synchrotron XRD refinements, one can observe rocksalt peaks that remain sharp while the spinel‐like peaks arising from the spinel cation superstructure ordering are broad. This suggests that the extent of ordering is significant, but is limited to a very short coherence length. These are important features as the limited length scale over which spinel is coherent allows for the material to avoid the two‐phase reaction in well‐ordered spinel, and allows good rate capability over the lower 3 V feature.^[^ ^36^, ^62^, ^66^ ^]^ While the small secondary particles produced by milling with carbon produce good electrochemical performance, the damage done to the material complicates the characterization of *δ*‐DRX. Characterization of *δ*‐DRX at a larger (micron) particle size has recently allowed for an even greater understanding using a combination of scanning electron nano‐diffraction (SEND), synchrotron XRD, and HAADF.^[^ ^62^, ^65^ ^]^ The 3–7 nm spinel‐like domains that form in the material are found throughout the entirety of a micron‐sized given particle, with a 16c/16d disorder level obtained from the fitted synchrotron XRD patterns, such as those in Figure 7d, of 5–10%. These domains appear to nucleate, grow, and finally impinge on each other at antiphase boundaries formed between the different variants of the spinel lattice. These spinel variants form as a result of the symmetry reduction from cubic rocksalt lattice (Fm‐3 m) to that of spinel (Fd‐3 m). In contrast with PDS materials produced with ball‐milling, these materials appear to retain less disorder, form more distinct domain boundaries, and utilize significantly more Mn redox (less O redox). In summary, *δ*‐DRX is composed of partially disordered spinel domains with different spinel variants of 3–7 nm in size, which impinge on each other at antiphase boundaries.^[^ ^62^, ^65^ ^]^ No significant amount of DRX is left in the material.

For traditional DRX materials, a combination of Li‐excess, SRO, and particle size dictates the rate capability and energy content of a given compound. However, once spinel‐like order forms in a DRX, a large Li‐excess is no longer required to achieve percolation, enabling even higher Mn content. Li‐excess DRX cathodes with high Mn (Mn > 0.8 per O_2_) and low Ti content (ex: Li_1.05_Mn_0.85_Ti_0.1_O_2_) transform to *δ*‐DRX in the first 20–30 cycles, and this transformation is more extensive compared to that of materials with less Mn.^[^ ^36^, ^37^, ^38^, ^64^, ^119^, ^126^ ^]^ Formation of *δ*‐DRX during cycling provides several performance benefits including enhanced rate capability, better cycling stability, flatter voltage profiles, and higher specific energy. Because the spinel structure has a high fraction of 0‐TM channels, the high degree of spinel‐like order leads to greatly improved transport in these materials, the best of any yet‐reported thermally synthesized DRX.^[^ ^8^ ^]^ The cycling stability and voltage retention are also greatly improved by the large Mn redox reservoir and lack of appreciable oxygen redox, enabled by low Li‐excess. This unlocks some of the best capacity retention among all DRX‐derived materials, with some even showing almost no capacity or voltage fade during extended cycling.^[^ ^36^, ^65^ ^]^ The formation of plateau‐like features at around 3 V and 4 V versus Li/Li^+^ also benefits the energy content. The flatter voltage profile brought about by ordering allows for more capacity to be extracted within a given voltage window, leading to much of the energy content gain observed in these materials. As a result of the improved transport and flatter voltage profile, the capacity of these compounds observed in a window of 2.0–4.8 V versus Li/Li^+^ typically climbs from < 150 to over 200 mAh g^−1^, while the energy content grows from 400–500 to 650–750 Wh kg^−1^ upon transformation to δ‐DRX. The transformation therefore enables Mn‐rich DRX with specific energies which are competitive with that of NMCs. The combination of each of these traits makes them currently the subcategory of DRX compounds most likely to be commercialized first.

While the δ transformation creates a promising partially (dis)ordered material, made without mechanochemical synthesis, the transformation by regular galvanostatic cycling itself presents several challenges if completed in a fresh cell. The slow transformation will lead to poor initial cell performance and complicate the ability of battery management systems (BMS) to accurately assess SOC and balance cells. The 20 cycles required to transform the material take 3 weeks at the rate of 20 mA g^−1^ typically used in DRX literature, far too long to practically occur as the last stage of battery cell production. For δ‐DRX to be utilized in commercial cells, a method to transform the material prior to cell operation must be obtained, which has indeed been the focus of very recent research. Given the thermodynamic stability of spinel in delithiated Mn‐rich material, one option is to delithiate a material and heat it slightly to transform to spinel‐like order.^[^ ^62^, ^66^, ^141^ ^]^ This allows for the material to cycle with its maximum capacity and rate capability from the initial use of the cell, avoiding the slow transformation. This method also allows for the transformation of large particles, greatly simplifying electrode fabrication and improving cycling stability. Nonetheless, this approach would require partial chemical delithiation of DRX followed by mild heating and relithiation. Though this would complicate cathode fabrication, chemical delithiation is used at very large industrial scale in the fabrication of novel alkaline battery cathodes. Very recently, another method to transform to the δ phase, electrochemical pulsing has also been developed, using elevated temperature, high rate, moderate voltage “pulses” applied to the as‐fabricated cell to form δ‐DRX.^[^ ^65^ ^]^ The product is equivalent to material from cycling, and is remarkably stable. Comparison with cycled material reveals that it takes roughly 60–80 cycles or pulses for the transformation to completely stop. This technique allows for the transformation of 2–3 micron single crystals, which delivers close to 200 mAh g^−1^ between 2 and 4.8 V without voltage or capacity fade after 100 cycles.^[^ ^65^ ^]^ Given these successes, it is likely that these or other methods can be incorporated into material or cell manufacturing such that the first cycle after formatting utilizes an already transformed δ cathode.

## 7 Carbon

Compared to commercial Li‐ion cathodes (layered, olivine, and ordered spinel) which have been fine‐tuned over several decades, DRX cathodes are a nascent technology, and several processing challenges must be overcome to achieve competitive electrode‐ and cell‐level performance, as pointed out by Lee et al. in a recent review.^[^ ^6^ ^]^

Most reports on DRX cathodes utilize high carbon content to offset the active material's low electronic conductivity (e.g., 2 × 10^−7^ S/cm for DRX (Li_1.2_Mn_0.4_Ti_0.4_O_2_) vs 1 × 10^−3^ S/cm for LiCoO_2_)^[^ ^142^, ^143^ ^]^ and maintain interparticle connectivity during cycling. Furthermore, nonoptimal processing conditions for some materials in the DRX family (e.g., high energy ball‐milling) often yield heterogeneous electrodes due to material agglomeration and sedimentation. As highlighted in the following discussion, promising approaches to address these bottlenecks include: i) depositing thin, conformal carbon coatings onto DRX particles and/or (ii) incorporating nanostructured conductive additives with high aspect ratios to improve electrical connectivity.

**Figure 8:**
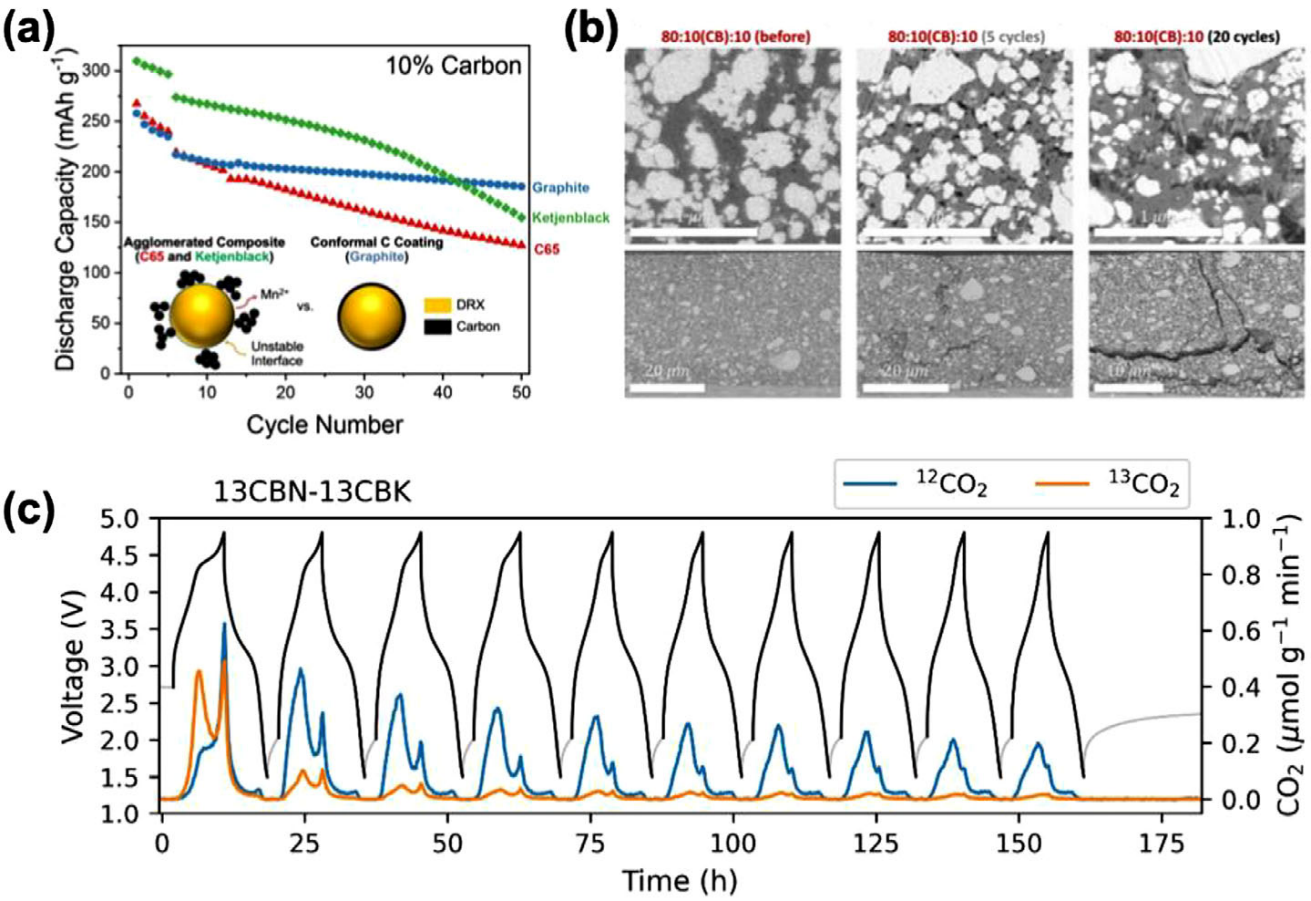
The Influence of Carbon on Electrode Stability and CO_2_ Evolution. a) Comparison between the capacity and cycling stability of electrodes fabricated with the same Li_1.2_Mn_0.5_Ti_0.3_O_1.9_F_0.1_ DRX material and carbon content, but three different types of carbon. Electrodes with graphite are found to have a lower rate of fade. Reproduced with permission.^[^ ^144^ ^]^ Copyright 2023, American Chemical Society. b) SEM imaging of an 80% active material, 10% carbon black, electrode in the pristine state, and after 5 or 20 cycles. Cracking and loss of contact between the PDS particles and the carbon additive are visible in the cycled samples and worsen from cycle 5 to cycle 20. Reproduced with permission.^[^ ^100^ ^]^ Copyright 2024, Royal Society of Chemistry. (=c) CO_2_ evolution from a Li_1.2_Mn_0.4_Ti_0.4_O_2_/Li cell with 1.0 M LiPF_6_ in EC, cycled between 4.8 and 1.5 V. The origin for the CO_2_ evolution is decoupled using isotopic labeling: CO_2_ originating from ethylene carbonate is shown in blue, CO_2_ originating from carbon black and residual lithium carbonate, both of which were 99% 13C labeled, is shown in orange. CO_2_ evolved from electrolyte degradation dominates after the first cycle. Reproduced with permission.^[^ ^154^ ^]^ Copyright 2024, American Chemical Society.

Relatively few systematic studies have reported how carbon additive selection impacts DRX performance. In recent work by Patil et al. shown in **Figure** **8**a, Mn/Ti‐based DRX cathodes (Li_1.2_Mn_0.5_Ti_0.3_O_1.9_F_0.1_) were prepared with three different conductive additives: carbon black (Super C65, 66 m^2^/g), Ketjen black (EC600JD, 1345 m^2^/g), and synthetic graphite (KS‐6, 17 m^2^/g).^[^ ^144^ ^]^ Scanning tunneling electron microscopy and Raman spectroscopy investigations showed that the high surface area carbons agglomerated during electrode processing, resulting in precarious electrical contact which was unable to accommodate cycling‐induced volume changes. On the other hand, mechanical exfoliation of graphite yielded conformal coatings on the DRX particles which enabled high‐capacity electrodes (e.g., 260 mAh g^−1^ at 10 mA g^−1^) with good cycling performance (85% capacity retention after 50 cycles). Zhou et al. reported similar benefits for disordered carbon coatings (*≈*10 nm thick, applied via milling) in Li_1.2_Mn_0.4_Ti_0.4_O_2_ cathodes containing 10 wt% carbon.^[^ ^145^ ^]^ Overall, these studies indicate that applying carbon coatings is one means to improve cycling performance while simultaneously reducing the weight/volume of inactive components.

Existing literature suggests that DRX oxide/oxyfluoride cathodes may be incompatible with carbothermal reduction or chemical vapor deposition routes used to apply carbon coatings, in contrast to other Li‐ion active materials (most notably LFP).^[^ ^146^ ^]^ For example, Xu et al. deposited amorphous carbon coatings on Li_1.2_Mn_0.4_Ti_0.4_O_2_ particles via thermal decomposition of acetylene.^[^ ^142^ ^]^ While this approach increased the cathode's electronic conductivity by 5 orders of magnitude (up to 1.4 × 10^−1^ S cm^−1^), the carbon‐coated DRX exhibited low initial coulombic efficiency (39%) and reversible capacity (70 mAh g^−1^). The authors attributed this poor performance to H^+^/Li^+^ exchange, which may have occurred during the coating process. It is also possible that the presence of carbon during heating damages Mn‐based DRXs by chemically reducing Mn^+3^ to Mn^+2^. To the best of our knowledge, a detailed study on carbon coatings derived from other organic precursors has not been reported for DRX cathodes, and DRX technology would significantly advance if a process could be developed to apply thin carbonaceous coatings to DRX particles without degrading the active material. Recently developed δ‐DRX may be particularly suited for such an approach as it can be used in single crystal form in the 1–3 µm range.^[^ ^65^ ^]^

Nanostructured conductive additives with high geometric aspect ratios represent another means to improve electrical connectivity and decrease carbon content in DRX cathodes. Lee et al. incorporated multi‐walled carbon nanotubes (MWCNTs) with Mn‐based DRX cathodes (Li_1.68_Mn_1.60_O_3.7_F_0.3_) and were successful in cycling electrodes with up to 96 wt% active material (*≈*5 mg DRX/cm^2^).^[^ ^100^ ^]^ In addition to providing robust electron transport pathways, the MWCNTs also improved the mechanical strength of laminates enabling low binder content (2 wt%). As shown in Figure 8b, it appears that much of the difficulty in achieving stable cycling at high energy density with a low content of carbon and binder is due to breaking contact between DRX particles and carbon. As shown by Lee et al. this problem can be mitigated by using carbon nanotubes.^[^ ^100^ ^]^ These cathodes exhibit impressive specific energies up to 1050 Wh kg^−1^ – the highest level reported to date at the electrode level. When cycled between 1.5‐4.8 V versus Li/Li^+^, the cathodes showed moderate cycling stability (65% capacity retention after 30 cycles at 25 mA g^−1^) and outstanding rate capability (244 mAh g^−1^ at 2 A g^−1^). In comparison, electrodes containing 10 wt% carbon black exhibited rapid capacity fade with only 11% retention after 30 cycles. The beneficial impact of carbon nanotubes (CNTs) has also been reported for other DRX chemistries including Li_1.24_Fe_0.38_Ti_0.38_O_2_ (LFTO)^[^ ^147^ ^]^ and Li_1.2_Mn_0.4_Ti_0.4_O_2_ (LMTO).^[^ ^142^ ^]^ Shen et al. added CNTs during the sol–gel synthesis of LFTO which yielded high capacity electrodes (226 mAh g^−1^ at 10 mA g^−1^) with improved rate capabilities (e.g., 80 mAh g^−1^ at 1.5 A g^−1^ versus 18 mAh g^−1^ for an electrode without CNTs).^[^ ^147^ ^]^ Similarly, Xu et al. showed that LMTO cathodes containing CNTs had higher capacity (165 mAh g^−1^) and improved cycling stability (73% capacity retention after 50 cycles) compared to an electrode prepared with carbon black (155 mAh g^−1^ and 28% capacity retention after 50 cycles).^[^ ^142^ ^]^

This initial research shows that significant gains can be made from the combination of DRX particle morphology engineering (e.g., through co‐precipitation,^[^ ^148^ ^]^ hydrothermal,^[^ ^149^ ^]^ and molten‐salt routes,^[^ ^150^ ^]^ and the judicious choice of carbon and its application to DRX electrode fabrication.

## 8 Electrolyte

Compared to conventional layered transition metal oxide (TMO) electrodes, DRX materials typically operate over a much wider cycling window (2.0–4.8 V vs Li/Li^+^ for DRX *cf*. 3.0–4.3 V vs Li/Li^+^ for NMC) to achieve similar specific energy^[^ ^151^ ^]^ and, unlike layered TMO cathodes, some DRX compositions rely on anionic redox as a significant part of their charge compensation mechanism, leading to the formation of reactive oxygen species (ROS). These two differences may introduce additional cathode‐electrolyte degradation pathways and require an electrolyte salt‐solvent system (and any decomposition products) to be stable over a much larger potential range and against ROS.

At the time of this review, only a few studies have explored the interaction between DRX materials and the electrolyte solution. These studies have primarily focused on Mn‐based DRX materials (Li─Mn─Ti─O(─F), Li─Mn─Nb─O(─F)),^[^ ^116^, ^119^, ^120^, ^121^, ^152^, ^153^, ^154^, ^155^, ^156^, ^157^ ^]^ and to a lesser extent Fe‐based (Li─Fe─Nb─O),^[^ ^158^, ^159^ ^]^ Ni‐based (Li─Ni─Ti─Mo─O(─F)),^[^ ^56^ ^]^ and V‐based (Li─V─O─F)^[^ ^160^ ^]^ compositions. As a result, the impact of the exact DRX composition, particularly the nature and relative content of the redox‐active element (Mn vs Fe vs Ni) on electrolyte degradation, has yet to be established. Among the various DRX compositions investigated, the older Li_1.2_Mn_0.4_Ti_0.4_O_2_ (LMTO2422) has been studied the most extensively. The following section will primarily address the findings related to this material while incorporating other compositions where relevant.

The electrolyte reactivity at the DRX interface for various compositions has been studied by characterizing the insoluble and gaseous electrolyte decomposition products formed during electrochemical cycling, using X‐ray photoelectron (XPS) spectroscopy,^[^ ^153^, ^158^, ^161^ ^]^ soft X‐ray absorption spectroscopy (XAS),^[^ ^116^ ^]^ solid‐state nuclear magnetic resonance (ssNMR) spectroscopy,^[^ ^116^ ^]^ titration mass spectrometry (TiMS)^[^ ^120^, ^121^, ^154^ ^]^ and differential electrochemical mass spectrometry (DEMS).^[^ ^120^, ^121^, ^152^, ^154^ ^]^

The as‐synthesized DRX surface of multiple compositions (Mn─Ti, Mn─Nb, Ni─Ti) contains trace quantities of Li_2_CO_3_ (*≈*1‐2 wt.%)^[^ ^56^, ^120^, ^121^, ^154^ ^]^ and LiF (in the case of fluorinated DRX),^[^ ^116^ ^]^ which remain from the synthesis. Li_2_CO_3_ can also form through exposure of DRX particles to ambient CO_2_, or during mechanical milling of the DRX particles with conductive carbon under inert atmosphere, where lithium and oxygen are extracted from the DRX particle oxidizing the carbon.^[^ ^154^ ^]^ The native Li_2_CO_3_ has been found to oxidize to CO_2_ on charge and partially reform during discharge, thereby exacerbating the interfacial electrolyte reactivity due to the known formation of reactive oxygen species such as superoxide and singlet oxygen during carbonate oxidation.^[^ ^154^, ^162^, ^163^ ^]^ Similar observations have been made for the Mn‐Nb DRX, Li_1.2_Mn_0.625_Nb_0.175_O_1.95_F_0.05_.^[^ ^116^ ^]^ Thus, the impact of impurities remaining from the synthesis procedure is not insignificant and can contribute to the interfacial reactivity and gas evolution at DRX electrodes. Alternative synthesis routes may lead to less reactive impurities to reduce the reactivity at the DRX interface.

While conventional carbonate‐based electrolyte solutions are nominally stable up to 5 V versus Li/Li^+^,^[^ ^164^, ^165^ ^]^ in operando gas measurements on Li_1.2_Mn_0.4_Ti_0.4_O_2_ half‐cells show the evolution of CO_2_, O_2_, and H_2_ gas during charging.^[^ ^152^, ^154^ ^]^ The formation of CO_2_ and H_2_ persists over 200 cycles, suggesting sustained reactivity at the DRX interface.^[^ ^152^ ^]^ Below, a detailed description of the gas evolution and the underlying electrode‐electrolyte processes is given.

On the first cycle, the CO_2_ evolution profile reveals interfacial reactivity starting at *≈*4.0 V (vs Li/Li^+^) and *≈*4.6 V (vs Li/Li^+^, note that this onset potential may be DRX‐composition dependent), the latter of which coincides with O_2_ and H_2_ evolution.^[^ ^152^, ^154^ ^]^ Notably, O_2_ evolution, which originates from surface DRX lattice oxygen oxidation, is only observed on the first cycle and has shown to be fully suppressed for fluorinated DRX materials, where Li extraction can be fully compensated by TM redox.^[^ ^119^, ^120^, ^121^ ^]^ On subsequent cycles, CO_2_ evolution starts immediately upon charging (*≈*1.5–2.0 V vs Li/Li^+^) and, along with the high‐voltage process, persists with little attenuation over 200 cycles, which indicates that carbonate species have to be forming persistently, possibly from an electrolyte or carbon originating reaction at high voltage.^[^ ^152^ ^]^ Similar outgassing behavior has been observed for Mn‐Ni based DRX (Li_1.2_Mn_0.6_Nb_0.2_O_2_ and Li_1.2_Mn_0.45_Nb_0.35_O_1.85_F_0.15_).^[^ ^120^, ^121^ ^]^

The low voltage CO_2_ evolution (*≈*4.0 V) is attributed to oxidation of the surface carbonates. Using isotopic labelling of the native carbonate species, it was uncovered that CO_2_ evolution on the first cycle predominantly originates from lithium carbonate decomposition (Figure 8c).^[^ ^154^ ^]^ Lithium carbonate decomposition at these potentials has been shown to occur through electrochemical oxidation,^[^ ^162^, ^166^ ^]^ but a contribution from an acid‐base mechanism may occur at higher potentials.^[^ ^167^, ^168^, ^169^ ^]^

The high‐voltage CO_2_ gas evolution (*≈*4.6 V) mostly originates from decomposition of the carbonate solvent, with a minor contribution from carbon oxidation.^[^ ^154^ ^]^ The reported electrochemical stability window for the various carbonate solvents typically used is >5.5 V versus Li/Li^+^,^[^ ^164^, ^166^ ^]^ and thus alternative mechanisms beyond electrochemical oxidation have been considered. Drawing on insights from conventional layered TMO electrodes,^[^ ^170^, ^171^, ^172^, ^173^ ^]^ solvent decomposition has been attributed to a chemical oxidation mechanism involving reactive lattice oxygen species. In the case of layered transition metal oxides, a small amount of singlet oxygen has been detected from NMC electrodes and is proposed to be involved in chemical oxidation reactions.^[^ ^172^, ^173^, ^174^ ^]^ For DRX materials, oxidized lattice oxygen species have also been detected through TiMS,^[^ ^56^, ^155^ ^]^ yet the exact nature of these oxidized lattice oxygen remains unknown. Density functional theory (DFT) also supports the formation of peroxo‐like species in DRX due to the presence of Li‐O‐Li configurations.^[^ ^67^ ^]^ Recent studies using DFT calculations have shown that the reaction between singlet oxygen and ethylene carbonate is slow,^[^ ^175^ ^]^ and unlikely to explain the extensive CO_2_ evolution observed through DEMS measurements. Instead, the authors identified peroxide species (O_2_^2−^) and superoxide species (O_2_^1−^) as the likely culprits for chemical oxidation of carbonate solvents.

Finally, additional low‐voltage CO_2_ evolution (*≈*1.5–2.0 V) is observed only after the first charge–discharge cycle and when discharged below 2.0 V.^[^ ^152^ ^]^ This suggests that this process may be coupled to a reductive process occurring on the preceding discharge. For example, the carbonate solvent may be reduced to a solid, carbonate‐like species, which is then re‐oxidized in the subsequent charge cycle. However, the exact nature of this species remains unknown.

To reduce the interfacial reactivity, several strategies have been employed, such as switching electrolyte salt‐solvent systems,^[^ ^158^, ^159^, ^161^ ^]^ incorporating electrolyte additives,^[^ ^176^ ^]^ fluorine incorporation,^[^ ^7^, ^119^, ^120^ ^]^ and employing surface coatings.^[^ ^156^, ^157^, ^160^ ^]^ Localized high‐concentration electrolytes (LHCEs) showed improved capacity retention compared to conventional electrolytes. Improved stability of the anion redox in Li_1.13_Mn_0.66_Ti_0.21_O_2_ was observed when cycling in an electrolyte consisting of LiFSI in EC/DMC with a diluent of 1,1,2,2‐tetrafluoroethyl‐2,2,3,3‐tetrafluoropropylether (TTE)^[^ ^161^ ^]^ The capacity retention improves from 19.9% using LiPF_6_ in EC/DMC (1:2 by wt) to 72.5% after 200 cycles between 2–4.8 V. XPS shows that when TTE is added to the electrolyte with LIFSI a stable and uniform LiF‐rich cathode‐electrolyte interphase (CEI) forms. This inorganic‐rich interface has lower electronic conductivity compared to organic interfaces, preventing further decomposition of the electrolyte and yielding a thinner CEI.^[^ ^161^ ^]^ In addition, nanosized Li_1.14_Mn_0.57_Ti_0.29_O_2_ in high concentration LIFSI in DMC (molar ratio of 1 : 1.1) paired with an aramid‐coated polyolefin separator enhanced the capacity retention from 57% to 73% and coulombic efficiency from 98.5% to 99.2% after 50 cycles compared to using LiPF_6_ in EC : DMC = 3 : 7 (vol%).^[^ ^177^ ^]^ The polar groups in the aramid improve the wettability of the high concertation LIFSI. The combination of HCE and an aramid‐coated polyolefin separator have been argued to prevent side reactions and TM dissolution while maintaining great wettability.^[^ ^177^ ^]^ Ionic liquids (ILs) have also been investigated as electrolytes for DRX materials. When an electrolyte of 0.5 M LITFSI in N‐propyl‐N‐methylpyrrolidinium bis(trifluoromethylsulfonyl)imide (Pyr1,3 TFSI) was paired with Li_1.25_Fe_0.5_Nb_0.25_O_2_ it was possible to achieve 72% capacity retention and 99.6% CE after 100 cycles between 1.5 and 4.6 V. XPS and gas chromatography showed no formation of LiF or Li_2_CO_3_ on the surface and minimal gas evolution during cycling.^[^ ^159^, ^160^ ^]^ In addition, ILs suffer from aluminum dissolution from the current collector, and small amounts of ethylene carbonate (*≈*10%) are still required for the formation of an effective solid‐electrolyte interphase (SEI) on the negative electrode.^[^ ^158^, ^159^ ^]^ The incorporation of F into Mn‐Nb‐based DRX materials has been shown to limit the extent of anionic redox and suppress gas evolution (O_2_ and CO_2_) during the first charge‐discharge cycle.^[^ ^119^, ^120^, ^121^ ^]^ However, no significant improvement in the outgassing behavior has been detected after the second cycle. A recent DFT study further supports that fluorine doping reduces oxygen loss.^[^ ^77^ ^]^ Alumina and borate coatings have also demonstrated an improved electrochemical performance.^[^ ^156^, ^157^, ^160^ ^]^ To improve the compatibility between DRX and electrolytes, strategies which have been effective with other cathode systems can potentially also be applied to DRX.^[^ ^6^, ^182^, ^183^ ^]^ These include tuning the fluorination degree of ether‐based solvents to improve the stability window of electrolytes,^[^ ^178^, ^179^, ^180^ ^]^ designing asymmetric ether solvents to achieve a high transport rate,^[^ ^181^ ^]^ and methylation of electrolytes or addition of additives such as 1,3,6‐hexanetricarbonitrile to promote inorganic LiF/Li_3_N formation at the interfaces.^[^ ^6^, ^182^, ^183^ ^]^

## 9 Remaining Issues

**I) DRX electrochemical stability**: DRX represents a chemically diverse and growing set of high energy cathode materials with many compounds made using only Earth‐abundant TMs. Given the pressing need for an energy‐dense replacement for Ni‐ and Co‐based materials, we now consider the remaining practical challenges to the commercialization of DRX. For most applications, a given electrode system must be capable of attaining a cell lifetime of at least *≈*1000 cycles to be commercially viable. Many early DRX compositions have struggled with capacity fading due to the excessive use of oxygen redox from high Li‐excess level.^[^ ^45^, ^58^, ^67^, ^88^, ^105^, ^128^ ^]^ This issue is common to many Li‐rich materials at high voltage.^[^ ^184^, ^185^, ^186^ ^]^ The associated lattice reconstruction and TM dissolution, observed in some DRX materials (ex: Li_1.2_Mn_0.4_Ti_0.4_O_2_), would be a significant issue in full cells with graphite anodes where Mn reduction at the anode can prevent the formation of a stable SEI layer.^[^ ^145^, ^187^ ^]^

Given that many of these stability issues originate from the reliance on oxygen redox, we anticipate a shift toward Mn‐rich compositions. This is because if minimal O‐redox is desired, and thermal synthesis with minimal F incorporation is used, then a Mn content of 0.7‐0.8 is required to achieve 210–240 mAh g^−1^ of Mn^+3^/Mn^+4^ redox. For DRX with high Mn content, no evidence of O‐redox in DEMS and improved cycling is observed.^[^ ^36^, ^37^, ^62^, ^113^ ^]^ This shifts the design space of interest mainly to those compounds which transform to δ‐DRX. While electrolyte decomposition is also responsible for degradation of DRX cathodes, it is exacerbated by the oxidation of oxygen, TM dissolution, and a wide voltage window during cycling, which are all less problematic for the δ‐DRX system. Therefore, because of their high energy and high intrinsic stability, the δ‐DRX group of materials is a strong candidate for future commercialization. The rest of our discussion will therefore mainly focus on δ‐DRX.

**II) Synthesis of Mn‐Rich DRX**: The shaker‐milling used to reduce DRX particle size of some older DRX compositions is energy intensive and often necessitates 10–20 wt% carbon to coat the large surface area created by particle size reduction. The large surface area of these DRX compounds and carbon degrades cycle life relative to that of larger particles, as it provides additional surfaces for side reactions.^[^ ^154^ ^]^ As such, this practice should ideally be avoided. Direct synthesis of the cathode materials at a particle size tolerable with the relatively slow diffusion in DRX composition is therefore desirable.

To remain above the cation disorder temperature, Mn‐rich DRX requires high synthesis temperatures (> 1000 °C), which can lead to rapid particle coarsening. In molten salt synthesis, DRX particles grow to 3–5 µm after only 20 minutes at 1100 °C.^[^ ^65^ ^]^ Coarsening in solid‐state synthesis is not significantly slower, but is less homogeneous, producing large particles along with submicron particles.^[^ ^88^, ^188^ ^]^ Shorter heating times may be necessary to produce 1 µm particles that can be used without milling. In addition to rapid particle growth at high temperature, Mn‐based DRX are typically synthesized in an argon environment. The susceptibility of these materials to oxidation necessitates furnaces with an inert atmosphere, which must be flooded with argon (or potentially nitrogen) prior to heating. At larger batch sizes, slower heating rates will lead to more time spent at or near the synthesis temperature, regardless of the length of the hold. While these challenges may be fundamental for high temperature synthesis, they also may be readily solved by developing rapid synthetic techniques.

Because solid‐state synthesis requires pelletization before and pulverization after synthesis, we argue in favor of a modified molten salt synthesis. This method allows for control of particle size, and the minimization of surface area by the use of spherical single crystals. Notably, Wu et al. have recently reported a microwave‐based approach to synthesize several‐micron DRX (in air) in 5 minutes, suggesting rapid thermal synthesis methods are possible and may alleviate the need for Ar.^[^ ^84^ ^]^ We see precise particle size control from a rapid and scalable synthetic method as one of the most important outstanding challenges for producing viable δ‐DRX cathodes. In the case of PDS and other materials made by mechanochemical synthesis, we believe that thermal methods to produce these materials are essential for practical application.

**III) Particle Size, Diffusivity, and Rate Capability**: Based on some initial reports, the diffusivity of δ‐DRX (roughly 10*^−^* ^14^ cm^2^ s^−1^)^[^ ^65^ ^]^ is 2–4 orders of magnitude lower than that of Ni‐rich NMC (10*^−^* ^12^–10*^−^* ^10^ cm^2^ s^−1^).^[^ ^189^, ^190^, ^191^, ^192^ ^]^ Based on the characteristic diffusion time for a spherical particle, NMC811 is expected to have a reasonable rate capability for *≈*5 µm single crystals, but δ‐DRX will likely require single crystals below ≈1 µm to attain similar performance. The diffusivity of δ‐ DRX is comparable to LFP (LFP has 1‐D diffusion channels, but its diffusivity can be approximated as an apparent 3‐D diffusivity),^[^ ^193^ ^]^ which has been commercialized despite also being a poor electrical conductor.^[^ ^194^, ^195^ ^]^

**Figure 9:**
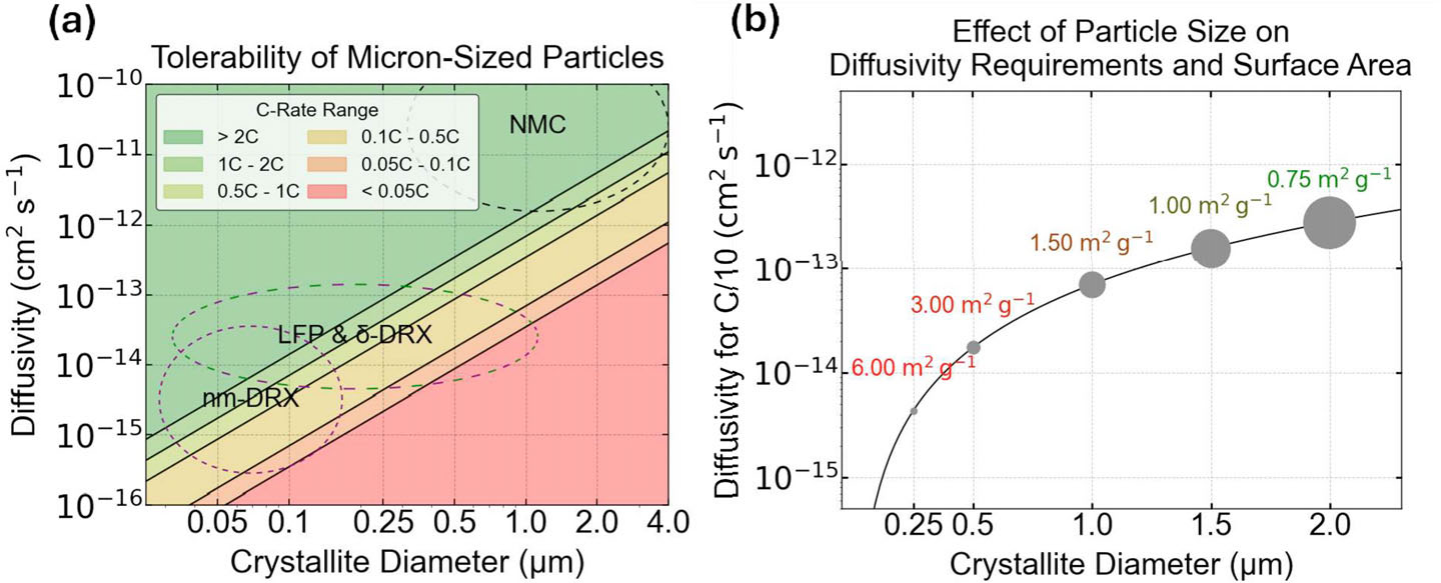
The interplay of diffusivity, particle size, and electrode surface area. a) Mapping of the C‐rates attainable by cathode materials of a given diffusivity and spherical particle diameter. b) The effect of particle size on the diffusivity required to tolerate a C/10 discharge rate and the surface area of the cathode material.

It should also be noted that many of the diffusivity values for NMC and LFP in the literature may be inaccurate due to their unaccounted‐for secondary morphology, meaning their particle diameters are not interchangeable with δ‐DRX single crystals. As such, we utilize only approximate diffusivity values from GITT on single crystalline NMC, LFP, and DRX as a comparative estimate of particle size requirements. **Figure** **9**a shows the approximate diffusivity required at various particle sizes for a discharge at various C‐rates. For fixed discharge time, the necessary diffusivity grows with the square of the particle radius. Established vehicle and grid‐scale storage use cycles, suggest that for most applications, Li‐ion cells need only be capable of *≈*2 or 3C rates on charge and ≈1–2 C at the end of discharge.^[^ ^196^, ^197^ ^]^

As can be seen, to achieve a discharge rate of 2C with a 1 µm particle size, a diffusivity of 10*^−^* ^13^–10*^−^* ^12^ cm^2^ s^−1^ is necessary. Alternatively, if submicron particles are obtained, diffusivity in the range of 10*^−^* ^14^–10*^−^* ^13^ cm^2^ s^−1^ may be tolerable, approximately the range already achieved by δ‐DRX. Some other DRX compositions, without spinel‐like order, which achieve only 10*^−^* ^15^–10*^−^* ^14^ cm^2^ s^−1^ would require direct synthesis at a particle size on the order of 100 nm. The surface area of a sample of spheroidal particles decreases linearly with particle size when the mass is fixed. Given that electrolyte degradation is influenced by the surface area of the active material and carbon additive, it is desirable to minimize surface area by maximizing particle size. As shown in Figure 9b, particle sizes from 500 nm–1 µm are attractive due to their low surface area and good predicted rate capability (1‐2C). Here, the smooth single crystals formed at high temperature may be an advantage for reducing the surface area for side reactions. The use of 500 nm–1 µm single crystals would also simplify the challenge of reducing the carbon content of DRX electrodes, especially considering that thus far a successful conformal carbon‐coating of Mn‐based DRX, akin to the process used for LFP,^[^ ^146^, ^198^, ^199^, ^200^ ^]^ has not been achieved. We believe that developing a scalable method of creating 500 nm–1 µm single crystalline Mn‐rich DRX is critical for constructing energy dense and stable electrodes.

**IV) Carbon Content, Carbon Coating, and Voltage Window**: To produce high energy, long‐lasting full cells, the carbon content of DRX electrodes must be reduced to < 5 wt% and the coulombic efficiency must be > 99.95%. A low carbon content in electrodes has been demonstrated for ball‐milled materials,^[^ ^100^ ^]^ but must also be demonstrated for larger particle materials. If DRX single crystals are employed, cathode fabrication may be similar to NMC single crystals. Novel carbon coating approaches to apply thin graphitic‐like coatings on large‐particle DRX would be particularly beneficial for enhancing electrode‐level energy density, cathode stability, and electron transport. Such techniques need to be consistent with the chemical and thermal stability of the DRX cathode, which is currently not the case when industry techniques used for LFP cathodes are applied to DRX.

To achieve long cycle life, high coulombic efficiency must be maintained in tandem with high discharge capacity, meaning almost no electrolyte degradation should occur within the voltage range employed. This may be achieved by narrowing the cycling voltage window from 1.5–4.8 V to 2.0–4.6 V or 2.5–4.4 V versus Li/Li^+^. This would also eliminate some volume change at the end of discharge, and mitigate oxygen redox at the top of charge. Alternatively, electrolyte formulations stable within a wider window may also be used.

Given the different oxidative stability of the various alkyl carbonates, and the rise in attention to different Li salts such as TFSI, it is possible that a higher voltage electrolyte can be tailored to δ‐DRX. This may be accomplished using commercially available salts and solvents, potentially taking advantage of work done for high voltage LiNi_0.5_Mn_1.5_O_4_.^[^ ^201^, ^202^, ^203^ ^]^ Concentrated electrolytes have demonstrated stability within a wider operating window, though they have also proven to be expensive and corrosive.^[^ ^204^, ^205^, ^206^, ^207^ ^]^

Full cells containing DRX cathodes and graphite anodes must also be proven with practical loadings. The irreversible capacity during the first cycle is not well quantified for δ‐DRX but appears to be *≈*50 mAh g^−1^.^[^ ^37^, ^38^, ^65^ ^]^ This capacity loss may be attributed to several factors including: i) decomposition of residual Li_2_CO_3_ impurities, ii) electrolyte breakdown to form the CEI layer, and iii) extraction of Li^+^ at low voltage from the as‐synthesized DRX which is not retrieved when subsequent discharge is limited to 2 V versus Li/Li^+^. Some of this excess Li may prove useful for maintaining Li inventory and compensating for SEI formation, for which lithiation agents such as Li_6_CoO_4_ are sometimes used in commercial cells.^[^ ^208^, ^209^ ^]^ If this excess capacity exceeds that required for SEI formation or other losses such as Si additive lithiation, extra graphite will be required, which will lower cell energy density.

**Figure 10:**
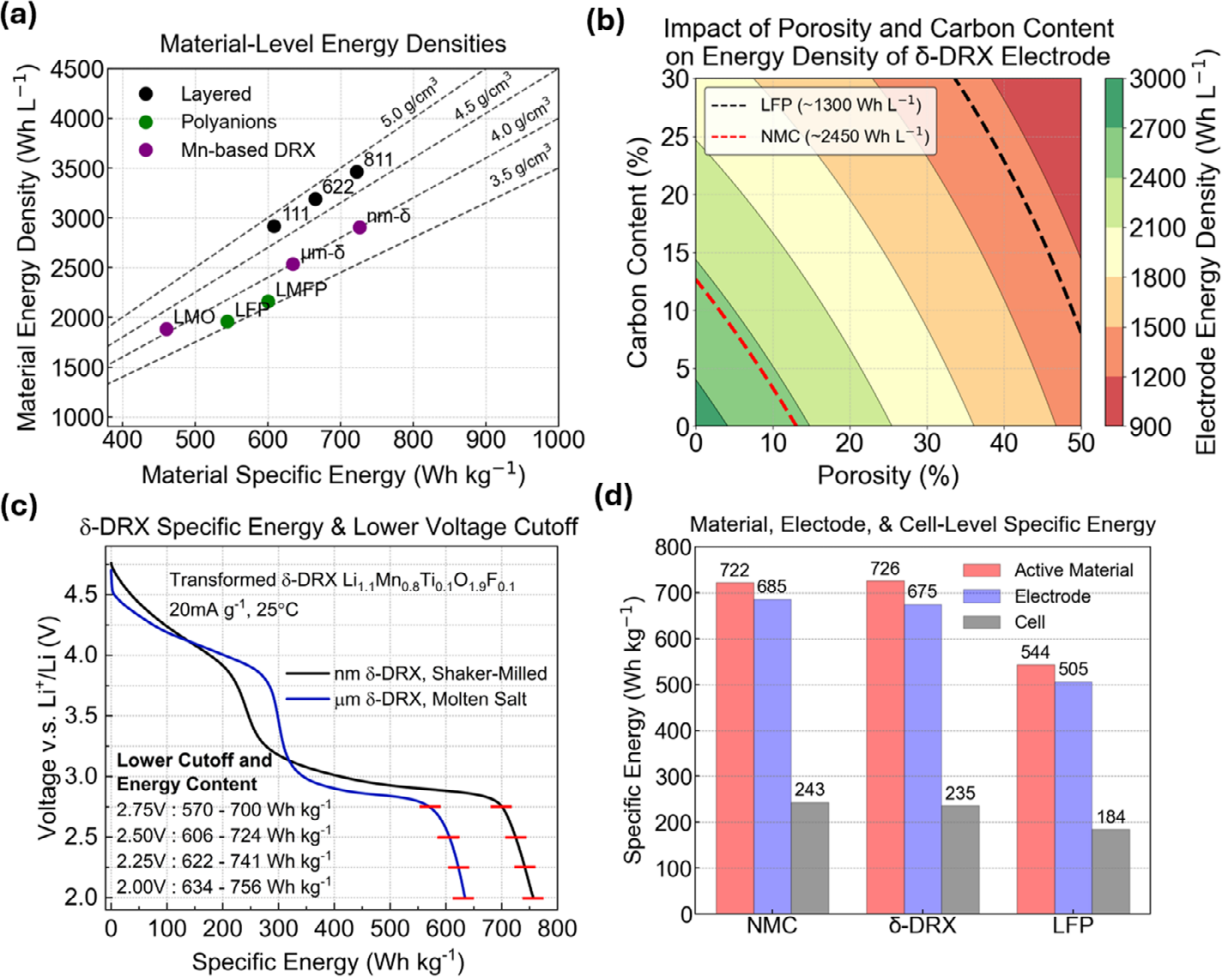
Attainable Energy Content of Delta Full‐Cells a) Energy density and specific energy of Mn‐rich DRX materials, the NMC series, LFP, and LMFP. b) The influence of electrode porosity and carbon content on the energy density of a δ‐DRX electrode, with lines designating the energy density of typical NMC811 and LFP cathodes. For simplicity, 20% porosity and 3 wt% binder is assumed. For LFP 30% porosity is assumed. c) Discharge voltage profile of fully transformed δ‐DRX at micron and nm particle sizes, showing the available specific energy within various voltage windows.^[^ ^65^ ^]^ d) Energy density at the active material, electrode, and cell level for NMC‐811, δ‐DRX, and LFP.

**V) Energy Density and Commercial Viability** Given recent results with Mn‐rich δ‐DRX, it is reasonable that these materials can achieve a stable cycling capacity of *≈*220 mAh g^−1^ at an average voltage of *≈*3.3 V versus Li/Li^+^. Assuming reasonable loadings, graphite anode, pouch cell, and other cell factors, we can estimate the electrode and cell level energy density and compare it with established cathode materials. **Figure** **10**a shows the attainable specific energy of δ‐DRX at the micron and nm level (*≈*700 Wh kg^−1^ AM) along with commercialized cathodes. Given the density of DRX (*≈*4.0 g cm^−3^) is between that of NMC (4.8 g cm^−3^) and LFP (3.6 g cm^−3^), these values translate to active material energy densities comparable to NMC at the material level.

Figure 10b shows the effect of the electrode film porosity and carbon content on the ability of δ‐DRX to surpass LFP and NMC. The ability of δ‐DRX to attain an electrode level energy density between LFP and NMC is critically dependent on achieving a *≈*3.0 g cm^−3^ cathode film, which requires a similar porosity (20%) to NMC cathodes. Additionally, the carbon and binder content must be reduced from the typical value of 20 wt% to less than 10 wt%, to decrease the mass and volume of inactive components. Thus far, we have assumed that the active material delivers the same capacity as that obtained in half cells cycled between 2.0 and 4.8 V versus Li/Li^+^. Figure 10c shows the energy density for currently available, δ‐DRX depending on the voltage cutoffs.^[^ ^65^ ^]^ At high voltage, almost no capacity is delivered above 4.4‐4.5 V versus Li/Li^+^, so limiting the upper cutoff to this point is reasonable. However, due to polarization, constant voltage holds may be necessary on charge to extract this capacity without going to higher voltage.

Granting that micron or submicron sized δ‐DRX delivers 220 mAh g^−1^ delivered between 2.5‐4.4 V, in an electrode with 3% carbon and 20% porosity, we can now estimate the cell‐level energy density. As shown in Figure 10d, using an adapted model for a typical automotive pouch cell,^[^ ^210^ ^]^ we find that the electrode level energy density of δ‐DRX translates to a cell specific energy of 235 Wh kg^−1^ and energy density of 546 Wh L^−1^, much closer to the values for NMC (243 Wh kg^−1^, 718 Wh L^−1^) than to LFP (184 Wh kg^−1^, 410 Wh L^−1^) obtained using the same model. This outcome relies on achieving a cathode loading of *≈*3.3 mAh cm^−2^ or 15 mg cm^−2^, much more than has currently been demonstrated, but similar to commercial NMC and LFP cells. If PDS or another Mn‐rich DRX material could be made at scale and crafted into such an electrode, it could achieve *≈*250 Wh kg^−1^ and *≈*600 Wh L^−1^ at the cell level, roughly matching NMC. To this end, important future directions include 1) demonstrating improved cycling within a constricted voltage window, 2) determining the severity of cross‐talk with graphite anodes, and 3) demonstrating dense electrodes with δ‐DRX, PDS, and other high energy DRX formulations, possibly with single‐crystal active materials. If these issues can be overcome, various DRX compositions might be commercialized as a more affordable, safe, and sustainable alternative to NMC in many higher energy applications.

## 10 Outlook

While challenges remain to the commercialization of Mn‐rich DRX materials, we believe that this class of materials is a very promising next‐generation cathode candidate. DRX cells have the potential to achieve an energy density comparable to the layered cathodes (LCO, NMC, NCA, Li‐rich) while also being as sustainable, affordable, and safe as polyanion cathodes (LFP and LMFP). Although Mn‐rich DRX still needs to prove it can attain a cycle life of >1000 cycles in full cells, there is reason for optimism. The Mn‐rich DRX which transform to the δ phase is significantly more stable than earlier prototypical Mn‐based DRX materials such as Li_1.2_Mn_0.4_Ti_0.4_O_2_, even without the large F content enabled by ball‐milling (ex: Li_2_MnO_2_F). Low Li‐ excess, and therefore a large reservoir of Mn to avoid oxygen redox, appears important for stability. In Mn‐based materials with no oxygen redox and no two‐phase reaction involving Jahn–Teller distortion, there is no clear materials‐level reason why stable cycling should not be possible.

Instability in other Mn‐rich materials, such as Li_1.2_Mn_0.6_Ni_0.2_O_2_, arises only when charging past the exhaustion of the Ni redox reservoir, after which oxygen is released.^[^ ^211^, ^212^ ^]^ Cycling below this threshold enables stable cycling in many Mn‐rich cathodes. To our knowledge, no other Mn‐based oxide cathode avoids both oxygen redox and the two‐phase spinel transition except Mn‐rich DRX, making it a uniquely promising candidate. Additionally, Mn‐based DRX may benefit in stability by being Ni‐free, as Ni is more catalytic to electrolyte decomposition than Mn.^[^ ^213^, ^214^, ^215^ ^]^ While Mn dissolution remains a concern, strategies such as carbon or oxide coatings, core–shell structures, and doping to increase Mn valence (e.g., with Mn^+4^) have been effective in other systems and could be applied here.^[^ ^36^, ^104^, ^128^, ^144^, ^216^ ^]^

Perhaps most critically, unlocking large, potentially single‐crystalline Mn‐rich DRX reduces surface area and should allow a much lower carbon content while limiting Mn dissolution and electrolyte breakdown. The isotropic cubic structure of DRX, along with spinel‐like ordering, may also help accommodate the larger volume changes of Mn^+3/+4^ redox compared to Ni and Co.^[^ ^51^ ^]^ Additionally, constraining the voltage window to 2.0‐4.6 V or even 2.5‐4.4 V would significantly reduce electrolyte breakdown, impedance growth, and Li inventory loss. Now that material‐level stability has been established, further research on electrolyte stability and full‐cell feasibility is needed. Nevertheless, we are optimistic that the challenges of Mn dissolution, electrolyte breakdown, and other barriers can be addressed, as they have been for NMC systems, where particle cracking, oxygen release, and anisotropic volume changes have all been mitigated.

Considering the vast amount of effort which has been devoted to optimizing the cycling stability of NMC materials, and the urgency with which next generation cathodes such as DRX are needed, high‐throughput computational and experimental methods may be helpful to accelerate development. For example, machine learning methods for the prediction of materials’ properties^[^ ^217^, ^218^, ^219^, ^220^ ^]^ and automated experimentation on synthesis and electrochemical testing^[^ ^221^, ^222^, ^223^, ^224^, ^225^ ^]^ may accelerate the understanding of the electrochemical performance and degradation modes in δ‐DRX. Additionally, many of the techniques developed and knowledge gained since NMC was discovered are now available for use of δ‐DRX. For example, to study the complex phase transformation from DRX to δ‐DRX, in‐situ XRD could be used to more rapidly understand the variables that control the domain size in δ‐DRX.^[^ ^226^, ^227^, ^228^, ^229^, ^230^ ^]^ For analysis of electrolyte stability, precise methods of coulometry and calorimetry can be applied,^[^ ^231^, ^232^, ^233^ ^]^ and potential Mn dissolution issues in full cells might be overcome given the learnings from LiMn_2_O_4_ full cells.^[^ ^234^, ^235^, ^236^ ^]^ Taken together, emerging high‐throughput computational and experimental techniques, along with the knowledge accumulated from established cathode materials may allow more rapid development of DRX materials.

Given its potential for high cell‐level energy density and long lifespan, DRX is an appealing chemistry to displace layered cathodes. Safety considerations further strengthen its appeal. Recent trends in EV battery design‐such as eliminating modules, using cells as structural members, and building larger cells‐require thermally stable chemistries. NMC cells, prone to oxygen release and thermal runaway, recently have begun to fail to outperform LFP at the pack level due to spacing requirements to prevent cascading failures. In contrast, LFP's thermal stability allows for larger cells and higher packing efficiencies, reaching 62% gravimetric and 85% volumetric efficiency.^[^ ^20^, ^237^, ^238^ ^]^ Mn‐based DRX is expected to achieve similar safety, as Mn^+4^ resists oxygen loss more strongly than Ni^+4^ (and Co^+4^) upon heating. This is due primarily to the lower energy of the Ni^+3/+4^ band, and its overlap with the top of the O_2_‐2*p* band, which facilitates electron transfer from oxygen to Ni^+4^ and oxygen release. As a consequence, Ni‐rich NMC can release oxygen below 200 °C, while LiMn_2_O_4_ and LiFePO_4_ do not until nearly 300 °C.^[^ ^239^ ^]^ It is reasonable to expect that the safety of DRX will be similar to LiMn_2_O_4_ and that the exothermic release of oxygen and subsequent oxidation of electrolyte which makes Ni‐based cells more dangerous than LFP should not occur in Mn‐based DRX. Oxygen release in prior DRX materials was tied to oxygen redox, which suggests that both are minimal or absent in Mn‐rich δ‐DRX, as has been confirmed by mapping of resonant inelastic X‐ray scattering (mRIXS) and DEMS.^[^ ^36^, ^38^, ^62^, ^113^ ^]^

**Figure 11:**
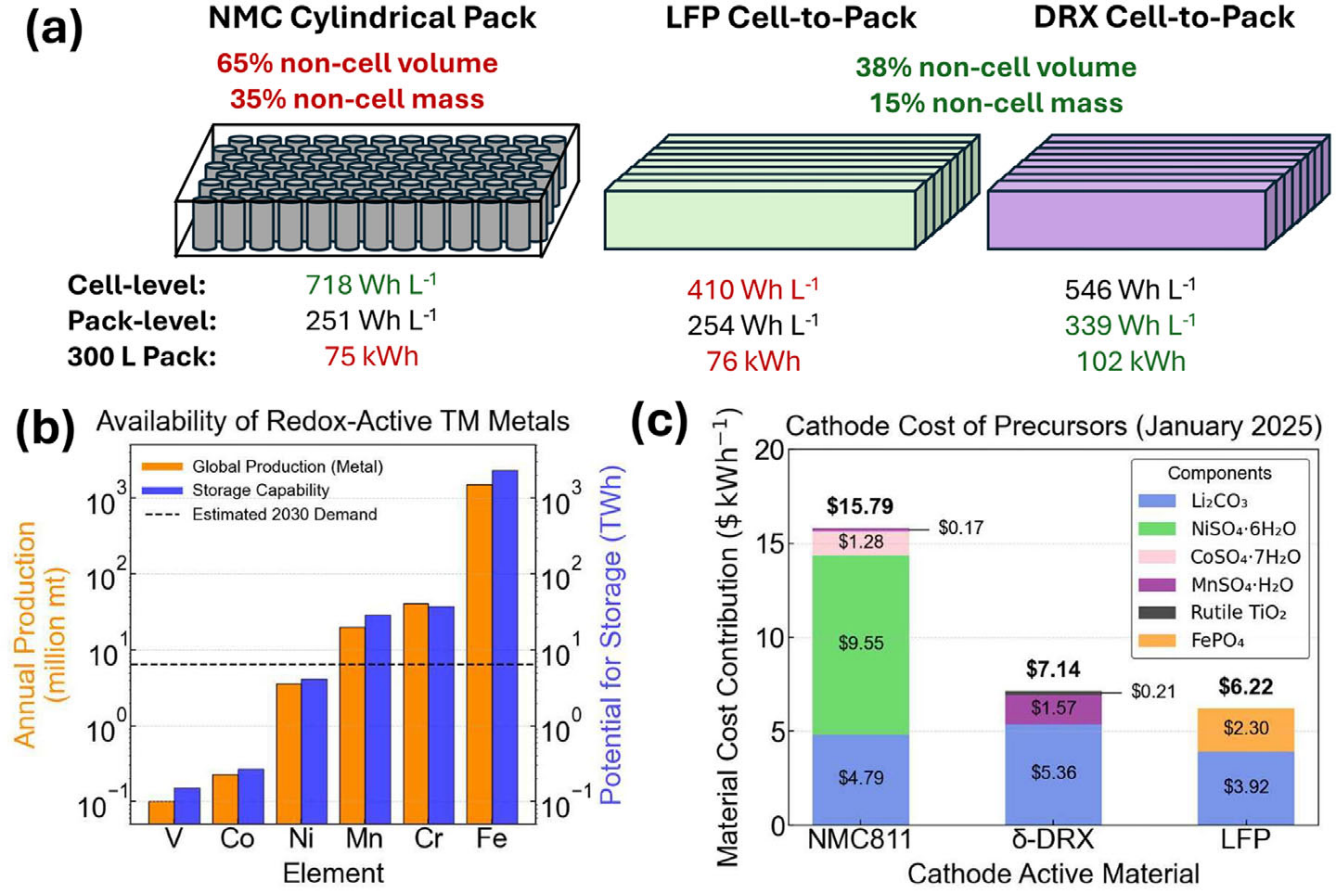
Attainable Energy Content of Delta Full‐Cells. a) The effect of cell‐to‐pack efficiency on the size and mass of the battery required to construct a 60 kWh EV battery at the cell and pack level. b) The global production volume (2024) of the most abundant redox‐active transition metals (orange)^[^ ^241^ ^]^ and the potential of these metals for energy storage with LIBs (blue), assuming an energy density of 700 Wh kg^−1^ for the compositions of Li_1.2_V_0.8_O_2_, LiCoO_2_, LiNiO_2_, Li_1.05_Mn_0_._85_Ti_0.1_O_2_, and LiCrO_2_, and 544 Wh kg^−1^ for LiFePO4 c) Materials cost breakdown for NMC811, δ‐DRX, and LFP based on January 2025 Shanghai Metals Exchange pricing of battery‐grade, key constituent precursors.^[^ ^134^ ^]^

Assuming that δ‐DRX can be used in a cell‐to‐pack configuration similar to LFP, this could grant it a significant advantage at the pack‐level, relative to NMC and NCA. As shown in **Figure** **11**a assuming the 62% and 85% gravimetric and volumetric cell‐to‐pack ratios achieved for the BYD Han EV,^[^ ^20^, ^240^ ^]^ δ‐DRX could achieve pack‐level specific energy and energy densities of 193 Wh kg^−1^ and 328 Wh L^−1^, well beyond NMC's value of 164 Wh kg^−1^ and 218 Wh L^−1^. This indicates that even if DRX always has a lower energy density than NMC at the cell level, it could easily surpass it at the pack level, while also satisfying the safety and cost requirements of EVs and grid‐scale storage installations. Looking forward into the future of this broad class of materials, solving the challenges for PDS or other higher energy Mn‐rich DRXs would lead to materials with higher energy than NMC at the cell, and far surpass it at the pack level.

The Earth‐abundance of Mn and Ti (and Cr) as compared with Ni and Co suggests that DRX materials will face fewer supply constraints and have the potential to be very low cost. The annual production of Mn, *≈*20 million tonnes, could supply 29 TWh/year, or roughly 30 times 2024 Li‐ion battery production Figure 11b.^[^ ^242^ ^]^ In contrast, current Ni production is *≈*3 million tonnes,^[^ ^4^ ^]^ which is mostly consumed by the steel industry. This could only supply *≈*4.1 TWh/year of battery production (assuming no constraint from cobalt, and *≈*700 Wh kg^−1^ for an NMC). Even if all nickel currently produced globally could be redirected to battery production, this would not be sufficient for the estimated 6.5 TWh of worldwide demand for batteries in 2030.^[^ ^2^ ^]^ Additionally, proven reserves of nickel would last only 40 years, at the current (insufficient) rate of production.^[^ ^241^ ^]^ While reserves grow for all commodities in lockstep with production as new resources are discovered, Ni faces additional challenges. Ni production from newer sources in Indonesia (which has almost half of known reserves) and elsewhere are much lower‐grade and associated with much higher CO_2_ emissions intensity than class I nickel sources in Canada, Russia, and elsewhere.^[^ ^4^, ^19^, ^243^ ^]^ As a result, it is likely that Ni, especially lower‐carbon nickel desirable to the consumer‐facing battery industry, will continue to be expensive, while Mn price will not be greatly affected by battery production for some time, and Fe likely never will be. As a consequence of the issues with Ni production, lower energy density LFP cells must support a significant and increasing fraction of Li‐ion production until a viable, energy‐dense Mn‐based alternative can be commercialized.

As shown in Figure 11c, taking current (January 2025) Shanghai Metals Market spot prices for the relevant Li, Mn, Ti, Ni, Co, Fe, and P precursors into account, the cost of materials for δ‐DRX (Li_1.05_Mn_0.85_Ti_0.1_O_2_ comes out to $7.1 kWh^−1^.^[^ ^134^ ^]^ This is significantly lower than our current estimate for the materials cost of layered cathodes like NMC811 ($15.8 kWh^−1^) and is comparable to LFP ($6.2 kWh^−1^). Several other considerations may impact real prices. First, the high synthesis temperature of DRX may require more expensive furnaces or more energy. However, the rapid synthesis of DRX, in minutes instead of hours, may counteract this to a large extent. DRXs also use Li less efficiently (*≈*0.59 kg Li_2_CO_3_ kWh^−1^) than LFP (*≈*0.43 kg Li_2_CO_3_ kWh^−1^) or NMC (*≈*0.53 kg Li_2_CO_3_ kWh^−1^). In this sense, DRX would be more price sensitive to Li than LFP. However, the very high Li spot prices needed before Mn‐rich DRX is more expensive at the raw material level than NMC are not likely to occur or be sustained because they would surpass the breakeven point of many currently non‐economic brine, mineral, and clay Li sources.^[^ ^241^ ^]^ Other production costs, such as lower costs from carbon coating and the higher manufacturing costs for less energy dense cells, may realistically mean that δ‐DRX cells come out at prices similar to LFP cells. If this is the case, then δ‐DRX cells could play a very large role not only as the ideal chemistry for cell‐to‐pack application in EVs, but also in grid‐scale storage where cost and thermal stability matter most. Therefore, we believe that stable δ‐DRX cells would have the potential to be a dominant cathode chemistry, displacing not only NMC based on cost and safety, but also LFP based on energy density, from a wide variety of applications.

In summary, the discovery of DRX materials has opened up a large and growing field of work on disordered (and partially disordered) cathode materials, which were once assumed to be electrochemically inactive. With Li‐excess, SRO manipulation, and even partial ordering, cathodes with extraordinarily high energy density can be made with no distinct diffusion channels. While the composition space of ordered oxides and polyanion cathodes are limited by the need to maintain distinct diffusion channels, the design space of DRX is widened to nearly all TMs. From this space, Mn‐based DRX cathodes have emerged as a large composition subspace with compounds that can match or surpass the energy density of state‐of‐the‐art Ni based layered cathodes, at a fraction of the material cost. These materials can be fluorinated to a higher extent than other oxide cathodes, possess SRO which modifies their transport properties, and can be made by a variety of thermal synthetic methods. The additional discovery that these materials transform to a spinel‐like phase beneficial to Li transport brings them closer to commercial viability. The reduced reliance on Li‐excess leads to less oxygen redox and capacity fade, while the improved diffusivity unlocks micron‐sized particles. These large particles will greatly simplify composite electrode design with low carbon loading. Mn‐based δ‐DRX materials have the characteristics needed to be a workhorse of transportation electrification and grid‐scale storage. While Ni‐rich layered cathodes have high energy density but poorer safety and material scarcity issues, and LFP is safe, reliable, and cheap but not energy dense, δ‐DRX has the potential to have better attributes of both. The challenges that remain with material optimization, electrode design, and electrolyte stability appear surmountable. Due to the rapid growth of the Li‐ion industry, and the lack of other Ni‐free cathodes of comparable energy density, it is important that Mn‐based δ‐DRX be prepared for commercialization as rapidly as possible. If the challenges discussed above can be overcome, the stalling energy density gains and material scarcity of existing cathode systems can be sidestepped and the electrification of transport and the expansion of grid‐scale storage for intermittent renewable energy storage can proceed rapidly.

## 11 Conclusion

Over the past decade, a diverse and growing design space of DRX materials has emerged as compelling candidates for next‐generation cathodes for Li‐ion batteries. These materials combine high energy density, Earth‐abundance, low‐cost, and safety‐critical factors for large‐scale applications in electric vehicles and grid energy storage. Despite challenges such as optimizing scalabe synthesis techniques for submicron particle sizes, achieving low carbon content in electrodes, and enhancing cycle life, recent advances in material design, synthesis methods, and electrode engineering indicate that these obstacles are surmountable. The unique properties of Mn‐rich δ‐DRX, including their high specific energy, good cycling stability, and safety, position them to rival and potentially surpass existing Li‐ion cathode technologies. As efforts to refine these materials continue, further discoveries of and improvements to these materials are expected to drive a more rapid, sustainable, and affordable energy transition.

## Conflict of Interest

The authors declare no conflict of interest.
